# Non-invasive prognostic protein biomarker signatures associated with colorectal cancer

**DOI:** 10.15252/emmm.201404874

**Published:** 2015-08-07

**Authors:** Silvia Surinova, Lenka Radová, Meena Choi, Josef Srovnal, Hermann Brenner, Olga Vitek, Marián Hajdúch, Ruedi Aebersold

**Affiliations:** 1 Department of Biology, Institute of Molecular Systems Biology, ETH ZurichZurich, Switzerland; 2 Institute of Molecular and Translational Medicine, Faculty of Medicine and Dentistry, Palacký UniversityOlomouc, Czech Republic; 3 Department of Statistics, Purdue UniversityWest Lafayette, IN, USA; 4 Division of Clinical Epidemiology and Aging Research, German Cancer Research Center (DKFZ)Heidelberg, Germany; 5 German Cancer Consortium (DKTK), German Cancer Research Center (DKFZ)Heidelberg, Germany; 6 Department of Computer Science, Purdue UniversityWest Lafayette, IN, USA; 7 College of Science and College of Computer and Information Science, Northeastern UniversityBoston, MA, USA; 8 Faculty of Science, University of ZurichZurich, Switzerland

**Keywords:** colorectal cancer, prognostic protein biomarker, targeted proteomics

## Abstract

The current management of colorectal cancer (CRC) would greatly benefit from non-invasive prognostic biomarkers indicative of clinicopathological tumor characteristics. Here, we employed targeted proteomic profiling of 80 glycoprotein biomarker candidates across plasma samples of a well-annotated patient cohort with comprehensive CRC characteristics. Clinical data included 8-year overall survival, tumor staging, histological grading, regional localization, and molecular tumor characteristics. The acquired quantitative proteomic dataset was subjected to the development of biomarker signatures predicting prognostic clinical endpoints. Protein candidates were selected into the signatures based on significance testing and a stepwise protein selection, each within 10-fold cross-validation. A six-protein biomarker signature of patient outcome could predict survival beyond clinical stage and was able to stratify patients into groups of better and worse prognosis. We further evaluated the performance of the signature on the mRNA level and assessed its prognostic value in the context of previously published transcriptional signatures. Additional signatures predicting regional tumor localization and disease dissemination were also identified. The integration of rich clinical data, quantitative proteomic technologies, and tailored computational modeling facilitated the characterization of these signatures in patient circulation. These findings highlight the value of a simultaneous assessment of important prognostic disease characteristics within a single measurement.

See also: **S Surinova *et al*** (September 2015)

## Introduction

Oncomarkers used in the clinic have a major impact on cancer detection, stratification into distinct subtypes, effective therapy selection, or outcome prediction. Reliable and easily measurable biomarkers are intensely sought after to enable a more personalized patient management (Ludwig & Weinstein, 2005; Surinova *et al*, 2011). Prognostic biomarkers are associated with survival that is independent of the therapeutic effect (Cunningham *et al*, 2010). Carcinoembryonic antigen (CEA)—the only FDA-approved biomarker in colorectal cancer (CRC)—is the marker of choice for monitoring the response of conventional therapy and detecting disease recurrence (Locker *et al*, 2006; Duffy *et al*, 2007). Additional and alternative prognostic and predictive biomarkers are urgently needed to improve the current clinical procedures in CRC. Stage at diagnosis, as determined by the TNM (tumor, node, metastases) classification system, is the most important prognostic factor. Patients diagnosed with localized disease (stages I and II) have an excellent 5-year survival rate of 90.1%. However, prognosis worsens rapidly with advancing stage where patients diagnosed with a regional spread (stage III) and metastatic disease (stage IV) have a 5-year survival rate of 69.2 and 11.7%, respectively (Siegel *et al*, 2012). Therapy selection is mainly driven by stage, yet groups of patients that do not benefit from the given therapies remain. One of the key clinical questions in CRC therapy is which patients to treat with chemotherapy among the stage II and III patients because some, but not all, will benefit from the aggressive and costly treatment (Brenner *et al*, 2014). In this area, added predictive value for survival beyond stage could be particularly beneficial. An improved prognostic stratification could thus offer more tailored therapeutic decisions for these patients. Hence, a non-invasive assessment of prognostic tumor characteristics together with an improved outcome prediction at diagnosis represents an important clinical goal toward a more effective management of CRC patients.

Large-scale genomic and proteomic profiling platforms are key technologies that allow us to comprehensively map molecular alterations associated with distinct clinical features and disease subtypes. For example, a recent integrated proteogenomic study of CRC tumors provided a functional context to previously reported genomic profiles (Cancer Genome Atlas, 2012), and proposed protein-driven subtyping of patient tumors, by prioritizing genomic alterations with the largest effects on the protein level (Zhang *et al*, 2014). The study produced a discovery-driven catalogue of protein-level alterations, and a functional characterization of the tumor biology of CRC.

This manuscript presents a complementary approach, which examined the prognostic significance of biomarker candidates in the circulation with respect to the patient’s clinical records. By using mass spectrometry-based discovery proteomic analysis, we discovered 303 glycoproteins that changed in abundance between tumor and adjacent normal epithelia of CRC patients (Surinova *et al*, 2015). Furthermore, using targeted mass spectrometry, we determined that 80 of these protein biomarker candidates could be reproducibly quantified in plasma of these patients (Surinova *et al*, 2015). This list of initially proposed biomarker candidates in plasma was used to quantify the respective proteins in suitable patient cohorts. The results were used for the generation of predictors of 5-year overall survival, and other clinicopathological characteristics that influence disease outcome. We discovered a six-protein biomarker signature for the prediction of patient outcome. With this signature, we were able to stratify prognosis beyond clinical stage and identified groups of patients with a high and low risk of the disease. The outcome signature was also found to correlate well with the corresponding transcript level profiles in additional independent cohorts of patients. Further, we evaluated the predictive ability of the signature in the context of other transcriptional signatures that recently attempted to redefine CRC subtypes and associated these subtypes with prognosis. Here again, our signature was able to predict these subtypes and rank them according to their prognosis.

Additionally, we explored whether the plasma protein data could be used for the prediction of other clinically relevant characteristics of CRC and found biomarker signatures predicting regional disease localization and metastatic dissemination. These predictions were further evaluated in independent cohorts, where feasible, on the protein level and, in both cases, on the transcript level. Interestingly, shared proteins between the biomarker signatures were observed, such as between the outcome and the metastasis signatures, suggesting that different prognostic CRC characteristics may be functionally interlinked at the molecular level. The newly identified biomarker signatures propose potential non-invasive blood-based alternatives to the current tissue biopsy-based methodologies and their performance warrants their further clinical evaluation in a prospective cohort of subjects with CRC.

## Results

**Figure 1:**
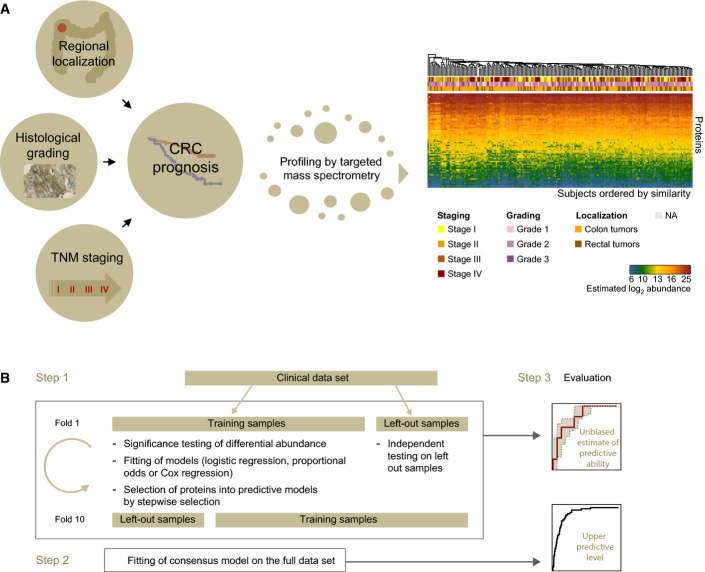
The development of biomarker signatures for the prognosis of CRC Comprehensive clinicopathological features of CRC included regional disease localization within the colorectum, histological grading, and TNM staging classification. Five-year overall survival was used as the main indicator of CRC prognosis. Targeted mass spectrometry based on selected reaction monitoring (SRM) was used to monitor CRC biomarker candidates in plasma and lead to the generation of a quantitative protein dataset. Subjects were ordered by similarity of their protein profiles and annotated with clinical data.

To identify novel prognostic biomarkers measurable non-invasively in the blood circulation of CRC patients, we designed a clinical cohort to reflect the major clinical and disease characteristics of the target population (Table1). In total, 202 patients were selected. The cohort roughly comprised an equal number of cases per clinical stage (stage I: *n* = 43, stage II: *n* = 58, stage III: *n* = 49, stage IV: *n* = 52). The clinical and molecular features of the cohort represented an inherent distribution that is typical for CRC. This cohort comprehensively characterized CRC, in that it included patients with a broad spectrum of regional localizations of cancer, TNM stages, and histological grading, and was annotated with overall survival with a follow-up of 8 years (Fig1A).

**Table 1:** Clinical and molecular characteristics of the colorectal cancer cohort

		Total	TNM stage			
			I	II	III	IV
*n*		202	41	58	51	52
Gender	Female/male	89/113	20/21	28/30	19/32	22/30
Median age at DG in years (25–75% quantiles)		67 (59–74.75)	64 (59–73)	68.5 (62.25–75)	66 (54–74)	65.5 (57.75–73.25)
OS	Median OS in years	8.8 (6.3–n.a.)	n.a.	n.a.	8.9 (n.a.)	2.0 (1.2–2.9)
RFS	Median RFS in years	n.a.	n.a.	n.a.	n.a.	2.3 (1.1–4.3)
KRAS	mut/wt	64/117	13/22	16/41	18/32	17/22
Microsatellite stability	MSI-high posit/total	25/173	5/33	9/56	7/48	4/36
	MSI-low posit/total	42/173	5/33	7/56	18/48	12/36
	MSS posit/total	106/173	23/33	40/56	23/48	20/36
Grading	G1	32	11	8	4	9
	G2	132	26	37	35	34
	G3	30	4	12	9	5
T	1	13	13	0	0	0
	2	39	28	0	5	6
	3	118	0	58	41	19
	4	9	0	0	5	4
N	0	104	41	58	0	5
	1	73	0	0	51	22
M	0	150	7	6	7	0
	1	52	0	0	0	52
RG DG	C18 + C19	131	19	41	31	40
	C20	71	22	17	20	12

DG, diagnosis; OS, overall survival; RFS, relapse-free survival; mut, mutated; wt, wild-type; MSI, microsatellite instability; MSS, microsatellite stability; posit, positive; G, grade; T, tumor; N, node; M, metastasis; RG DG, regional diagnosis.

The dataset was deployed for the development of biomarker signatures able to predict the respective clinical endpoints. Biomarker candidates were selected into the signatures within 10-fold cross-validation. Within each fold, the two criteria for selection were differential protein abundance between clinical groups and their subsequent stepwise selection into predictive models. Subjects were then classified and the procedure was evaluated on the left out samples. The procedure was repeated for each fold, and a consensus model was derived from the most frequently selected proteins across all the folds. An unbiased performance is derived from the pseudomedian fold of the cross-validation (i.e. between fold median) and 25^th^ and 75^th^ percentile bounds are also reported. An upper level of performance is reported for the consensus model on the full dataset.

We used targeted mass spectrometry based on selected reaction monitoring (SRM) to profile biomarker candidates (Appendix Table S1) in plasma with the goal to identify biomarker signatures (i.e. combination of multiple proteins) associated with and able to predict the above outlined clinical endpoints of CRC. Details of candidate biomarker identification and their high-throughput quantitative profiling across clinical cohorts are described in Surinova *et al* (2015). Briefly, 88-plex candidate measurements were performed simultaneously on the plasma *N*-glycoproteome of the CRC patients. These high-throughput measurements lead to the generation of a dataset in which 88 proteins were quantified over 202 CRC patients (Fig1A).

This quantitative dataset was deployed to discover associated biomarker signatures with clinical records that hold prognostic value of disease outcome. Overall survival was our primary clinical endpoint. We also considered several additional endpoints: regional localization, histological grading, and TNM staging (individual stages and metastatic state).

The discovery of biomarker signatures was performed using Cox proportional hazard regression (for the survival endpoint), logistic regression (for binary endpoints, e.g. regional localization), and proportional odds regression (for endpoints with multiple ordered categories, e.g. grading). For each endpoint, the selection of a subset of proteins with predictive ability was done by 10-fold cross-validation (Fig1B). For each 9/10^th^ of the patients, the eighty quantified protein candidates were first tested for significant differential abundance between groups. Proteins meeting this initial criterion within the fold were then employed as candidate predictors and used for a further stepwise protein selection into a predictive model within the same fold. The predictive ability of the selected model was evaluated using the remaining 1/10^th^ of the patients. The same procedure was repeated 10 times, by systematically selecting different 9/10^th^ and 1/10^th^ of the patients. Finally, a consensus model was formed from the most frequently selected proteins in all folds, that is, proteins selected in at least five of the ten folds.

We evaluated the predictive ability of the models as follows. For the overall survival endpoint, the sensitivity and specificity of predictors were summarized in a ROC curve based on the Cox model (Heagerty & Zheng, 2005). For the binary endpoints, the sensitivity and specificity of predictions were summarized in single ROC curves (Fawcett, 2006). For the endpoints with multiple categories, the sensitivity and specificity of predictors were summarized in individual ROC curves of all possible category pairs. For each of the methods, first, the summaries were obtained for the ten models discovered in each of the ten folds, using the patients in the left-out validation subset. These estimates are unbiased, and approximate the real-life performance of the selected consensus model. The predictive ability is summarized with AUC_median_, and calculated as a pseudomedian over the left-out datasets in all the folds. Second, these summaries were obtained for the consensus model on the full dataset. Since a large proportion of these samples participated in selecting the predictive proteins, these results are optimistic and can be viewed as an upper bound of the true performance. The predictive ability is summarized with AUC_full_. Third, to check the robustness of the results to the partition of the patients into the folds, the procedure was repeated using eight-fold cross-validation, and this led to the selection of a similar subset of proteins with comparable performance characteristics (Appendix Tables S3, S5 and S9). Fourth, we evaluated the association of clinical factors, such as age, gender, and TNM stage, with the clinical endpoints studied. The clinical factors were included in the predictive models by forced inclusion. With the exception of the survival endpoint, where clinical factors are known outcome predictors, proteins selected into all other predictive models were reproducible with the biomarker candidates selected into models without clinical factors, as was the performance of both sets of models, suggesting that these clinical factors do not play a substantial role for the clinical endpoints in our cohort (Appendix Table S10).

### Survival and patient outcome

**Figure 2:**
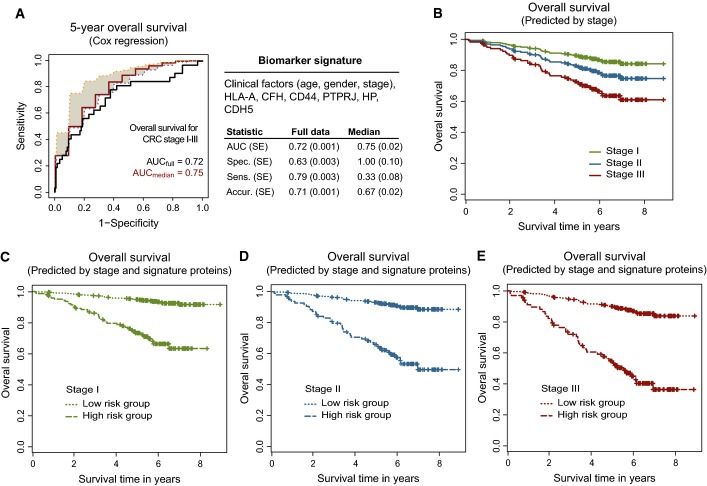
Biomarker signature of CRC outcome A Biomarker signature containing clinical factors and biomarker candidates predicting 5-year overall survival. The summary statistics obtained on the cross-validated pseudomedian validation fold (i.e. between fold median; labeled in red), corresponding 25^th^ (in magenta) and 75^th^ (in orange) percentile bounds, and on the full dataset for the consensus model (i.e. biomarker signature; labeled in black). SE was calculated by bootstrap (see methods) for full dataset and from the ten folds for the pseudomedian. SE, standard error; spec., specificity; sens., sensitivity; accur., accuracy.

Clinical factors—age, gender, and especially stage—are currently employed in the clinic to assess patient prognosis. A predictor of patients’ outcome that combines biomarker candidates with known clinical factors is expected to enhance the discrimination between patients with a better or worse prognosis, and to thus assist in their clinical management. We therefore examined the association of biomarker candidates with patient survival, and generated models predicting patient outcome. The best signature for the prediction of 5-year overall survival consisted of the clinical factors (age, gender, stage), and of six biomarker candidates (HLA-A, CFH, CD44, PTPRJ, HP, and CDH5) (Fig2A, Appendix Table S2). The outcome of more than 70% of patients was accurately predicted. The areas under the ROC curve were AUC_full_ = 0.72 for the full dataset, and AUC_median_ = 0.75 for the cross-validated pseudomedian.

B–E All collected survival data were used to plot predicted survival based on the Cox model fitted with the following: (B) stage I, II, or III (Cox model: 0.018*age – 0.006*gender(1 = male, 0 = female) + 0.368*stage; fixed parameters: age = 68, gender = male, stage = I or II or III); (C) stage I and signature proteins; (D) stage II and signature proteins; and (E) stage III and signature proteins. The signature proteins represent a linear combination of protein intensities (0.739*HLA-A – 1.143*CFH + 0.811*CD44 + 0.334*PTPRJ + 0.398*HP – 0.869*CDH5). The cutoff of −0.037 used for prediction is the median of individual predictions for all patients in stages I+II+III. HIGHprot represents a high-risk group of patients with individual predictions ≥ cutoff and LOWprot represents a low-risk group of patients with individual predictions < cutoff. The Cox model used in (C–E): 0.018*age – 0.006*gender (1 = male, 0 = female) + 0.368*stage – 1.735*LOWprot; fixed parameters: age = 68, gender = male, stage = I or II or III; and LOWprot versus HIGHprot is plotted.

To assess the benefit of the proposed outcome signature as compared to the clinical outcome prediction standard (i.e. a model comprised from the clinical factors alone), the predictive ability of these two models was compared. The outcome model that included the signature proteins adjusted by the clinical factors explained the survival of patients better than the model that included the clinical factors alone (likelihood-ratio test, *P* < 0.0033). To inspect the contribution of the signature proteins to the predictive ability, we employed all the collected survival spanning more than 8 years of observations and visualized the model-based predictions of probability of survival for each stage independently. These predictions were obtained with the following models: (i) the Cox model with fixed stage to I, II, or III, age and gender to median; and (ii) the Cox model with fixed stage to I, II, or III, age and gender to median, and a linear combination of the signature proteins. The predicted survival was inspected for the model without signature proteins (Fig2B, see Pseudocode of predictive analyses in Appendix for analysis details and Appendix Table S2C for model parameters) and the model including the signature proteins (Fig2C–E). A large separation of the predicted survival was observed for all stages pointing to an added value of signature proteins for outcome prediction and stratification of patients into prognostic groups. In addition to predicted survival, a stratified-survival visualization using Kaplan–Meier plots was also performed. Patients were stratified as above, into groups by stage alone (Appendix Fig S1A) or by the combination of stage and the signature proteins (Appendix Fig S1B–D). Again, a large separation of patients was observed and this was especially prominent for stage II and III CRC.

The discovered biomarker signature holds prognostic promise for newly diagnosed patients, because it can be measured non-invasively in blood plasma, and because it is associated with the survival. Notably, the added predictive value for survival beyond stage presents a potentially relevant substratification for treatment decisions.

### Transcriptional regulation of the outcome signature

To further characterize and evaluate the plasma protein outcome signature, its predictive ability was assessed on the transcriptional level. Two previously published independent datasets of adequate scope and scale were employed for this analysis. The first dataset GSE17536 from Smith *et al* (2010) contained 138 patients of TNM stages I–III, and overall survival (OS) was available with a follow-up of 12 years. The second dataset GSE14333 from Jorissen *et al* (2009) contained 139 patients of Dukes stages A–C, which roughly correspond to non-metastatic stages I–III of the TNM classification. Moreover, this cohort was associated with 5-year disease-free survival (DFS) with a follow-up of 12 years (as opposed to the overall survival used in our study). Even though the staging classification and the endpoint were somewhat different in this study, this cohort contained relevant prognostic associations for the evaluation of the outcome signature. Both datasets were acquired from tumor tissue samples of CRC patients on the HG-U133Plus2.0 platform, and both contained the transcripts coding for all six signature proteins. The transcript expression was employed as an indirect proxy of protein abundance. In both datasets, a Cox proportional hazards model was fit, using as predictors the transcripts corresponding to the signature proteins, and adjusted by the clinical factors. The parameters of the model were estimated by cross-validation, and the ability of the prognostic signature to predict OS or DFS was evaluated for the respective datasets. The resulting classifications were in the range of performance for the protein data (Appendix Figs S2A and S3A). Interestingly, a somewhat higher performance was obtained for DFS as compared to OS. To examine the performance of the signature genes individually, the parameters of a Cox model which used the transcripts as predictors (one predictor at a time) were estimated and the performance was reported for the full data and within cross-validation. The same procedure was also performed for the individual signature proteins in our proteomic dataset. When examining the areas under the ROC curves of individual proteins and genes, only CD44 and PTPRJ on the protein level and CFH on the transcript level (both for OS and DFS) showed higher AUC_full_ and AUC_median_ values than 0.6 (Appendix Table S11). This suggested that the two proteins and the CFH gene were the most important individual predictors of outcome. The enhanced multivariate prediction ability for DFS was not observed for the individual genes.

To evaluate outcome prediction beyond the current clinical standard on the transcript level, survival curves were plotted for individual stages predicted by clinical factors alone and by clinical factors and the signature genes. This has been done by analogy with the analysis performed on the proteomic data (as in Fig2B–E). Similar to the results on the protein level described above, there was a separation of patients into low- and high-risk groups for all stages, but this separation was more pronounced for stages II and III. This pattern was consistently observed for both transcriptomic datasets (Appendix Figs S2C–E and S3C–E), which supported the reproducibility of the improved patient stratification with the means of the outcome signature. These analyses determined that the outcome signature holds prognostic value also on the mRNA level.

### The outcome signature in the context of other prognostic signatures

Recent evidence from large-scale tumor tissue gene expression profiling suggests that classification of patients into subtype-specific groups helps to redefine prognostic signatures in CRC and can improve our understanding of CRC prognosis. Specifically, De Sousa *et al* (2013) characterized three molecularly distinct colon cancer subtypes (CCSs) in a cohort of stage II patients. Patients predicted to represent the CCS3 subtype demonstrated an especially poor prognosis. Another study by Sadanandam *et al* (2013) used a similar approach to discover five subtypes related to cellular phenotypes. Two of these subtypes (transit-amplifying and goblet-like subtype) showed good prognosis, two subtypes (inflammatory and enterocyte subtype) showed an intermediate prognosis, and the stem-like subtype demonstrated the worst prognosis. In both cases, gene expression signatures were proposed to predict these prognostic subtypes. We examined the overlap between signature transcripts identified in the two transcriptional signatures, and the proteins in the outcome signature presented here. Given that the gene expression profiling was carried out in tissue specimens, a large overlap between the transcript signatures and signatures derived from secreted glycoproteins detected in the circulation was not anticipated. The comparative analysis showed that CFH was the only molecular entity out of the six signature proteins that was also found in the 146-gene CCS signature. The 30-gene signature linked to distinct cellular subtypes had no overlap with our signature. This signature for the former comprised of 786 subtype-specific signature genes and was later condensed into the smaller 30-gene signature. When examining this initial gene set, CFH was again included in the signature. The occurrence of CFH in both transcriptional signatures further supports its regulation on the mRNA level, as was already suggested by CFH having the highest individual performance of all the signature genes on the transcript level (Appendix Table S11).

Next, we examined to what extent our outcome signature is able to predict the prognostic subtypes defined transcriptionally. For this analysis, the data used by the two respective studies were assembled and related to the protein data. De Sousa *et al* (2011) employed the GSE33113 dataset that was comprised of 90 stage II patients and the prognosis was associated with DFS. Sadanandam *et al* employed two datasets: GSE13294 (Jorissen *et al*, 2008) with 135 patients and GSE14333 (Jorissen *et al*, 2009) with 152 patients. Only GSE14333 data were annotated with prognostic data, that is, DFS.

**Figure 3:**
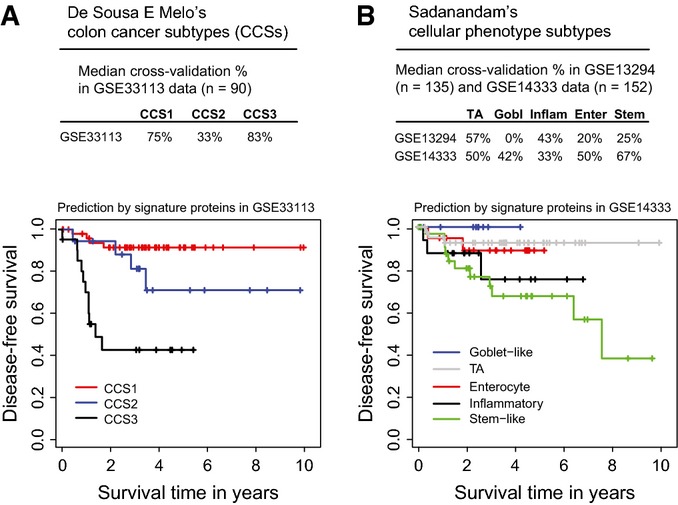
Prediction of transcriptional CRC subtypes A, B Proteins from the outcome biomarker signature were used to predict (A) three colon cancer subtypes (CCSs) in the GSE33113 dataset, and (B) five cellular phenotype subtypes in the GSE33113 and GSE14333 datasets. Kaplan–Meier curves were plotted for the respective subtypes based on the classification of the signature proteins. TA, transit-amplifying.

The predictive ability of the transcripts corresponding to the plasma protein signature was examined with respect to the three molecularly distinct colon cancer subtypes (CCSs) defined by De Sousa *et al* The GSE33113 dataset was used to estimate the parameters of a proportional odds model with the six gene proxies from our signature as predictors, and the 90 patients were classified within 10-fold cross-validation. The outcome signature was able to accurately predict 75% of the CCS1 cases, 33% of the CCS2 cases, and 83% of the CCS3 cases (Fig3A, see Appendix Table S12 for prediction tables from cross-validation). The results represent median percentages over the cross-validation folds. When plotting the Kaplan–Meier curves for patients belonging to the three subtypes as predicted by our classification, the obtained survival curves (Fig3A) were highly similar to the original curves obtained by De Sousa *et al* These results show that our outcome signature can predict a good prognosis and bad prognosis of CRC patients, that is, subtype CCS1 and CCS3, particularly well and that this prediction can be achieved using a minimally invasive procedure from the circulation by measuring six proteins.

Similarly, to predict the five prognostic subtypes related to cellular phenotypes defined by Sadanandam *et al* with the outcome signature, the parameters of a proportional odds model were retrained with the six gene proxies from our signature as predictors using cross-validation and classified the patient samples from the two datasets described above. The subtype that could be classified most accurately in both datasets was the transit-amplifying (i.e. good prognosis) subtype (Fig3B, see Appendix Table S13 for prediction tables from cross-validation). Additionally, in the GSE14333 dataset, the stem-like (i.e. bad prognosis) subtype could be predicted with a median correct classification percentage of 67%. Since GSE14333 contained DFS follow-up, the Kaplan–Meier survival curves were plotted again as predicted by the outcome signature and reproduced the outcome ranking (i.e. best to worst survival time) for all five subtypes.

The above analyses demonstrate that the outcome signature comprised from six proteins is able to predict, using non-invasive plasma protein measurements, transcriptionally defined prognostic subgroups recently characterized by two gene expression signatures.

### Regional tumor localization

Since CRC and its prognosis are influenced by a range of tumor characteristics, we next explored which other clinically relevant endpoints, in addition to outcome, could be predicted from our in-depth molecular data and assessed non-invasively.

The anatomical tumor localization in CRC is traditionally segregated in three basic anatomical sites: the colon (C18), the rectosigmoid junction (C19), and the rectum (C20). Other classification systems proposed alternative segmentation into proximal colon (i.e. right-sided colon proximal to the splenic flexure; C18.0-4), distal colon (i.e. left-sided colon distal to the flexure; C18.5-7+C19), and the rectum (C20) (Li & Lai, 2009). Patients with tumors from the different anatomic sites have been shown to have different patterns of survival, and generally, prognosis was better for colon than for rectal cancers. Moreover, colon and rectal cancers are also viewed as distinctive therapeutic entities. These differences were proposed to be due to their heterogeneity in accessibility, differential embryological origin, different functionality of the segments, and differences in molecular pathways driving these cancers (Li & Lai, 2009).

**Figure 4:**
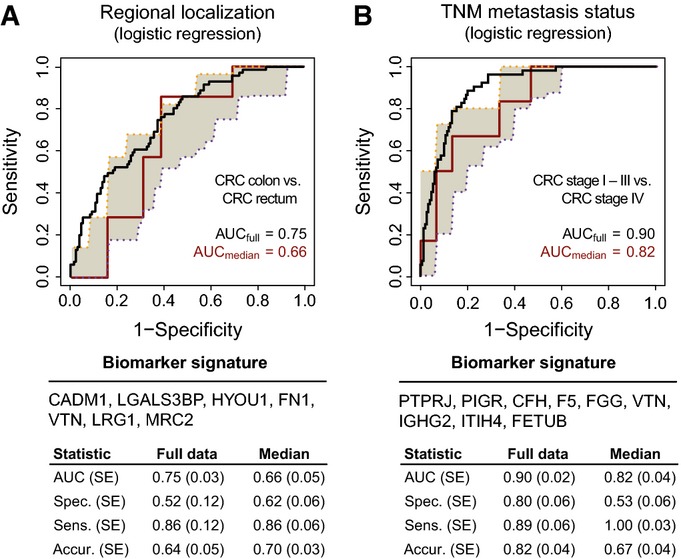
Biomarker signatures of additional prognostic CRC characteristics A, B Signatures for the prediction of (A) regional disease localization of colon (*n* = 131) and rectal (*n* = 71) tumors, and (B) localized (*n* = 150) and metastatic (*n* = 52) disease were also obtained. Summary statistics are represented as in Fig2, and for model parameters, see the Appendix. SE, standard error; spec., specificity; sens., sensitivity; accur., accuracy.

Non-invasive indicators of cancer localization within these segments may be clinically valuable as they can influence the sequence of interventions a gastroenterologist needs to perform to localize a tumor. We examined predictors for regional subtypes of CRC and also for colon and rectal cancers. The best predictor was obtained for the localization of colon (C18+19) and rectal (C20) tumors. The biomarker signature was comprised of seven proteins (CADM1, LGALS3BP, HYOU1, FN1, VTN, LRG1, and MRC2) (Fig4A, Appendix Table S4) that could predict the localization of rectal tumors especially well (86% of subjects with rectal cancer). The lower prediction accuracy of the colon tumor class (C18+19) may be attributed to the heterogeneity brought by having colon tumors as well as tumors at the rectosigmoid junction in the same group.

Next, we used an external proteomic dataset acquired by Zhang *et al* (2014) on 90 tumor tissue samples from the TCGA cohort (Cancer Genome Atlas N, 2012) for independent validation of this signature. Of these, 88 samples were annotated by regional localization (colon, *n* = 58, and rectal, *n* = 30, tumors). All seven signature proteins were also quantified by Zhang *et al* (2014). This dataset was obtained by data-dependent mass spectrometry and semi-quantification by spectral counting. Since the nature of spectral counting data is different from intensity-based SRM data, the parameters of the logistic regression model, which used the seven proteins as predictors, had to be estimated again in these data. The predictive ability of our signature to classify colon and rectal localization obtained on this new dataset was highly in accordance with the performance obtained in our data (Appendix Fig S4A).

Since the TCGA cohort had also RNA-seq measurements where transcripts corresponding to all signature proteins were measured, the level of concordance between the tissue transcriptomic and tissue proteomic classifications of the same patients could be directly examined. The parameters of the logistic regression model which used as predictors the genes corresponding to the signature proteins were estimated, and the ability of the localization signature to predict colon versus rectal cancer was evaluated. The obtained transcript classifications were similar but less accurate than those obtained on the protein level (Appendix Fig S4B). This trend was further confirmed on the complete TCGA cohort of 270 patients with 196 colon and 74 rectal tumors (Appendix Fig S4C).

With these results, the localization signature was validated on the protein level in an independent prospectively collected cohort of CRC subjects. Furthermore, an inferior performance was observed at the transcript level as compared to the protein level, proposing that the signature proteins are regulated to some degree posttranslationally. To investigate this suggestion for the individual signature proteins, the predictions were compared per protein in our dataset and in the complete TCGA cohort on the mRNA level, and confirmed that the classifications were more accurate on the protein than on the transcript level for six out of the seven proteins (FN1 showed a similar performance between the two platforms; Appendix Table S14).

### Histological grading

Tumor grade is a measure of cellular differentiation of tumor cells as compared to the normal cells in the tissue of origin. Histological grade is an important prognostic factor, independent of TNM stage. We attempted to identify proteins associated with histological grading. The proteins that were selected into predictive models within cross-validation varied markedly between the individual folds, and so did their predictive ability (Appendix Table S6). The observed performance may indicate that grading is a system too complex to be predicted with a handful of proteins or that molecularly diverse tumors are not identically classified, especially since the current grading system suffers from a significant inter-observer variability (Chandler & Houlston, 2008).

### Clinical stage and disseminated disease

Tumor assessment based on the TNM staging classification informs about the extent of the disease in terms of primary tumor invasiveness, regional lymph node spread, and the presence of distant metastases. At present, in the clinic an extensive and highly invasive procedure is used to develop patients’ treatment strategies and predict their prognosis. Being able to predict some of the aspects of the TNM system non-invasively would provide patients with a more acceptable solution. Based on the levels of secreted biomarker candidates, we searched for proteins that were able to predict TNM components.

Initially, we searched for a predictor of individual TNM stages (I, II, III, IV). More proteins were selected by differential testing and stepwise selection as compared to the other features. The final model included fifteen proteins (Appendix Table S7), nearly a double of the number of proteins selected into other biomarker signatures. The fact that the TNM system focuses on tumor invasiveness rather than size could have a major impact on the amount of biomarker secreted into the circulation and therefore may as well preclude an optimal predictor of TNM stage. An analogous observation was seen for stage-stratified CRC diagnosis (Surinova *et al*, 2015).

Next, we examined the prediction of disseminated disease (TNM stages I–III versus IV), as localized and metastatic diseases require different treatment strategies, and a non-invasive prediction of disease spread could be beneficial for the management of patients at diagnosis. A nine-protein signature (PTPRJ, PIGR, CFH, F5, FGG, VTN, IGHG2, ITIH4, and FETUB) was discovered and could predict the presence of metastatic or localized disease with an upper predictive level of AUC = 0.90 and with an unbiased predictive level of AUC = 0.82 (Fig4B, Appendix Table S8).

We further aimed to evaluate the dissemination signature on the set of TCGA samples that were measured by both proteomics and transcriptomics. Unfortunately, the metastatic group of samples was too small and precluded the reliability of the evaluation results. Hence, we examined the performance of the transcript proxies for our plasma protein signature directly on the full TCGA cohort with 224 localized and 40 metastatic tumor samples. The logistic regression model was retrained with all signature genes as above. The obtained classification results showed that on the mRNA level, the predictive ability of the signature was much lower than on the protein level (Appendix Fig S5). The metastatic signature is thus regulated to a smaller extent transcriptionally and to a much larger extent posttranslationally (as quantified by our data, Fig4B).

### Functional interplay between biomarker signatures of CRC

**Figure 5:**
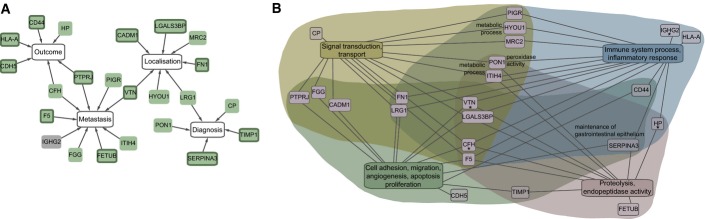
Relationship between signature proteins and their functional annotation Proteins from the biomarker signatures, including the diagnostic signature from Surinova *et al* (2015), were associated and interlinked by the proteins they have in common. Protein nodes shaded in green were previously associated with cancer and the ones with a highlighted border with CRC according to Ingenuity Pathway Analysis.

In summary, our results document measurable perturbations of CRC in the plasma proteome of patients and provide a concise list of proteins that are highly relevant for CRC due to their potential as prognostic biomarkers. We observed that the biomarker signatures of different endpoints often share one or two proteins (Fig5A). The graphical representation included the diagnostic signature from Surinova *et al* (2015). Although these proteins were not selected for their prognostic ability, we wanted to assess any overlap with the prognostic proteins. Indeed, LRG1 was observed as shared between the diagnostic and the regional localization signatures.

Protein annotation with gene ontology (GO) biological process terms. GO terms were summarized in four categories and connected to the respective proteins with a line. Additional terms of proteins belonging to the four main categories are labeled next to the respective proteins or with an asterisk in the case of the complement activation process.

Next, we examined whether proteins selected into the signatures were previously linked to cancer. For this analysis, associations of diseases with the signature proteins were searched in the Ingenuity Knowledge Base. Surprisingly, 22 of the 23 proteins that were part of at least one of the signatures developed in this study were linked to cancer (Fig5A, green protein nodes) and 12 of these were also associated with CRC (Fig5A, green protein nodes with a highlighted border).

To examine the functional interplay of proteins in more detail, the individual proteins were annotated with gene ontology (GO) biological process terms and the major associated processes were examined (Appendix Table S15). The identified processes were grouped into four categories: (i) cell adhesion, migration, angiogenesis, proliferation, apoptosis, (and maintenance of the gastrointestinal epithelium), (ii) signal transduction, transport, (and metabolic process), (iii) immune system process, inflammatory response, (and complement activation), and (iv) proteolysis, endopeptidase activity, (and peroxidase activity) (Fig5B). The processes in parentheses are applicable for specifically labeled proteins in Fig5B. The annotated processes are typically affected in cancer, which supports the functional involvement of the selected proteins in the biomarker signatures of CRC.

## Discussion

The present study was designed to develop plasma biomarker signatures for prognostic stratification of CRC, which would be comprised of a handful of proteins to facilitate their measurement with minimal invasiveness in a clinical setting. By focusing on the subproteome of glycoproteins, we identified biomarker candidates that were found in the circulation and could be reproducibly profiled with sensitive and multiplexed mass spectrometry-based methods using the targeting mass spectrometry technique SRM. The profiling of these candidates over a large clinical cohort led to the generation of a rich data source from which candidates associated with CRC endpoints could be extracted and used for their prediction. The clinical promise of biomarkers able to predict important clinical endpoints non-invasively is readily apparent, especially in the era where increasing efforts are directed toward tailored and preventive medicine.

A further promise of circulating as compared to tissue-based biomarkers comes from the nature of the tested material. In biopsy-reliant measurements, the biomarker refers to the respective small tissue area sampled from a larger tumor environment. In certain cases, a biopsy is taken from an area that contains more infiltrating immune cells than cancer cells, and a biomarker testing for a particular mutation will test negative for the sample, even if the cancer cells hold a mutation in the gene of interest. Given new insights from tumor heterogeneity analyses where not all lesions within a tumor were found to be identical (Gerlinger *et al*, 2012), it is important to sample several specimens or to employ an alternative readout that offers a summarized patient-level result from the circulation. This is especially relevant in CRC where numerous precursors (e.g. polyps) can be transformed into multiple malignant lesions. In the present work, the choice of profiling secreted proteins was guided by the aim to translate protein biomarkers from tissue to the blood circulation, and to facilitate non-invasive prognostic testing where a small set of markers is assayed from a blood sample.

Here, we report three biomarker signatures predicting CRC endpoints commonly assessed in the clinic by mainly invasive methodologies in tissue samples (i.e. colonoscopy and histology). These prognostic signatures were identified computationally employing cross-validation on the proteomic dataset to discover and evaluate their performance. To reach a real-life performance measure for multiple endpoints, it is particularly important to have cohorts with a large number of subjects, and ideally independent cohorts as were employed for the development of the diagnostic signature (Surinova *et al*, 2015). Such cohorts are difficult to obtain not only due to large experimental costs, but also because of the time it takes to attain a well-annotated cohort with clinical characteristics (especially survival data). Since this requires long-term planning, many cohorts lack such data. We report the predictive ability of the signatures on a large cohort with two readouts. First, an unbiased predictive performance is provided and is based on the untouched portion of the data during cross-validation of predictive models. This measure is independent of the signature discovery process and allows for an objective performance testing. Second, an upper predictive performance is reported for the consensus models of the biomarker signatures on the full data, which can be regarded as an optimistic approximation of the real-life predictive ability. Together, these two obtained performance readouts of the newly developed biomarker signatures warrant their prospective clinical evaluation in an independent clinical cohort. In the present work, the localization signature was externally validated on a prospectively collected cohort with proteomic measurements.

By far the clinically most relevant finding is the prognostic signature of outcome prediction. Currently, stage at diagnosis is the most important prognostic factor for CRC outcome. Although stage provides valuable prognostic information and guides therapy selection, the response and outcome of individual patients to a therapy is not predicted. With the prognostic biomarker signature, we highlighted an improved survival prediction and stratification of patients with a better or worse outcome, as compared to the analysis with stage alone. This was especially striking for stage II and III CRC. The patients with a high risk of death may likely represent individuals that ought to be treated as compared to the patients with a low risk of death that may not necessarily benefit from the given treatment. The prognostic signature and the improved informative markers therein could thus help to identify patients at high risk of relapse who might benefit from adjuvant therapy. From the six signature proteins (HLA-A, CFH, CD44, PTPRJ, HP, and CDH5), only CD44 has been previously associated with CRC prognosis in tumor specimens from 74 patients that were assayed by immunohistochemistry (Huh *et al*, 2009). Prognostic data for CD44 on the protein level measured in plasma across a sizeable cohort have not been shown before. However, our data indicate that the multivariate nature of the proposed signature contributes greatly to its performance as compared to the performance of an individual protein.

We have further evaluated the outcome signature on the transcriptional level in two independent datasets of adequate size (*n* = 138 and *n* = 139) that were associated with 12 years of survival follow-up data. Here, we used the transcript expression as an indirect proxy of protein abundance to estimate the predictive ability of the signature. Interestingly, the obtained predictions for the signature on the transcript level were in the range of the protein predictions. On the single gene level, we found that CFH held the highest accuracy as compared to the other genes individually. Its prognostic value in CRC may be related to recent reports, where complement factor H was found to be highly expressed in cutaneous squamous cell carcinoma (cSCC) (Riihila *et al*, 2014) and non-small cell lung cancer (NSCLC) (Cui *et al*, 2011) cells, and where it was associated with progression in cSCC and prognosis in NSCLC. In CRC, it was found to be part of a gene expression and pre-mRNA splicing signature that marks the adenoma-adenocarcinoma progression (Pesson *et al*, 2014) and in the recent transcriptional signatures defining new CRC subtypes (De Sousa *et al*, 2013; Sadanandam *et al*, 2013). We have also predicted overall survival and disease-free survival on the two mentioned transcriptomic datasets and were able to reproduce the large separation of patients into low- and high-risk outcome groups for stage II and III CRC.

Our findings are further in line with recent evidence from large-scale gene expression profiling, which suggests that classification of patients into subtype-specific groups can improve the understanding of CRC prognosis. Specifically, De Sousa *et al* defined an especially poor prognosis CRC subtype that is largely microsatellite stable (MSS) and contains relatively more CpG island methylator phenotype-positive carcinomas. Furthermore, this molecular subtype is refractory to anti-EGFR therapy (De Sousa *et al*, 2013). Highly similar gene expression profiles of these tumors facilitated the identification of this especially malignant CRC subtype, which could not be identified by characteristic mutations. Sadanandam *et al* used a similar approach to discover five subtypes associated with a differential response to classic chemotherapy and targeted therapies(Sadanandam *et al*, 2013). These subtypes could be related to different cells of origin in the colonic crypt, and gene expression signatures were proposed to identify these phenotypic subtypes. We have employed our outcome signature proteins to predict these respective subtypes on the transcript level in three different datasets and could classify especially well the subtypes of good (CCS1 & transit-amplifying subtype) and poor (CCS3 & stem-like subtype) prognoses. With this evaluation, we demonstrate that the outcome signature proteins hold value for indirect prognosis assessment based on newly defined CRC subtypes. The ultimate promise of reliable gene or protein signatures of prognosis comes from a subtype-specific patient stratification that may lead to a more effective management of this diverse disease.

## Materials and Methods

### Study population

The study was approved by the Ethics Committee of the University Hospital Olomouc and Faculty of Medicine and Dentistry, Palacky University, Olomouc, and all individuals have signed an informed consent document. Patients with colorectal cancer were selected consecutively at diagnosis. The sample cohort includes 202 patients (stage I: *n* = 43, stage II: *n* = 58, stage III: *n* = 49, stage IV: *n* = 52).

### Blood collection and plasma preparation

Blood was drawn prior to surgery from the cubital vein and collected into tubes processed with EDTA. Blood was directly centrifuged at 6,067 *g* for 3 min at 4°C. Plasma was collected into a new tube, frozen at −20°C, and stored at −80°C.

### Glycoprotein enrichment from plasma

Glycoproteins were isolated in a 96-well plate format as described in Surinova *et al* (2015). Briefly, glycoproteins were oxidized, and immobilized on resin, and non-bound proteins were thoroughly washed away with urea buffer (8 M urea, 100 mM ammonium bicarbonate, 0.1% SDS, 5 mM EDTA). Proteins were reduced with 5 mM dithiothreitol (DTT) at 25°C for 30 min and alkylated with 25 mM iodoacetamide (IAA) at 25°C for 45 min in the dark. Samples were diluted to 2 M urea, 0.025% SDS, 1.25 mM EDTA, and 100 mM ammonium bicarbonate and proteolyzed with sequencing grade porcine trypsin (Promega) at a protease to substrate ratio of 1:100, at 37°C for 15 h. *N*-linked glycosylated peptides were enzymatically released with *N*-glycosidase F at 37°C (PNGase F; Roche and New England Biolabs). Formerly glycosylated peptides were desalted in 96-well MacroSpin column plates filled with Vydac C18 silica (The Nest Group Inc.).

### Targeted LC-SRM analysis of plasma *N*-glycosites

Samples were analyzed as described in Surinova *et al* (2015) on a hybrid triple quadrupole/ion trap (4000 QTrap, ABI/MDS Sciex) equipped with a nanoelectrospray ion source and a Tempo NanoLC system (ABI/MDS Sciex) coupled to a 15-cm fused silica emitter, 75 μm diameter, packed in-house with a Magic C18 AQ 5-μm resin (Michrom BioResources). Peptides were separated over a linear gradient from 5% to 35% acetonitrile/0.1% formic acid over 35 min, at a flow rate of 300 nl/min. The instrument was operated in scheduled SRM mode (retention time window of 300 s, target scan time of 3 s), at a unit resolution (0.7 *m/z* half maximum peak width) of both Q1 and Q3 analyzers. SRM assays were retrieved from the *N*-glycosite SRM atlas (http://www.srmatlas.org/) (Hüttenhain *et al*, 2013), reanalyzed to select the best transitions for endogenous detection in plasma, and used to optimize a single SRM method. Internal standard peptides labeled with heavy isotopes at the C-terminal lysine or arginine, +8 or +10 Da, respectively, (Thermo Scientific, Sigma-Aldrich, or JPT Peptide Technology) were used to validate peptide identity by analogy of chromatographic and fragmentation properties to the reference. Raw data and SRM transition files can be accessed, queried, and downloaded via PASSEL (Farrah *et al*, 2012) from the SRMAtlas by following this link (https://db.systemsbiology.net/sbeams/cgi/PeptideAtlas/GetSELTransitions?SBEAMSentrycode=Crcpass2013) and selecting the validation dataset from the drop down menu of SRM experiments (Surinova_CRC_Biomarker_Plasma_Validation_Dataset, CRC).

### Relative quantification and statistical analysis of plasma *N*-glycosites

Raw data were processed as described in Surinova *et al* (2015). Briefly, files uploaded to MultiQuant 1.2 (Applied Biosystems) to perform automatic SRM peak integration and quantitative data were analyzed with MSstats (v.2.3.5) (Choi *et al*, 2014). Normalization was performed as described in Surinova *et al* (2015). Missing values were imputed for a given protein with a minimum summarization representing its limit of detection.

### Predictive analysis

Ten-fold cross-validation was used to find the most discriminative proteins. For each endpoint, subjects were divided into ten folds with equivalent proportions of a given endpoint as in the whole cohort. For each fold and for each endpoint, tests of differential abundance were conducted using MSstats (Choi *et al*, 2014), under the same settings as in Surinova *et al* (2015). For the survival endpoint, subjects alive at 5 years were compared to subjects with death up to 5 years, and censored subjects (*n* = 12) were ignored from testing analysis. Proteins with significantly differential abundance between groups were selected at FDR < 0.05 and fold change cutoff ± 1.1. MSstats was used to calculate the abundances of the proteins in each sample, on a relative log2-transformed scale that is comparable between runs. The relative abundances were used as input variables to logistic regression model (in the case of two groups), proportional odds model (in the case of more than 2 groups), and Cox regression model (in the case of survival data). In the case of Cox regression modeling, patients of stages I, II, and III were involved and the regression model was adjusted with clinical factors (age, gender, and stage). The best model for each fold in the training set was chosen by stepwise selection, which repetitively added or dropped proteins until minimizing Akaike information criterion (AIC). This best model was applied on the validation set in each fold. A final consensus model was derived for each endpoint from the ten respective models obtained within cross-validation and was comprised of proteins which were selected in at least five of the ten folds. To obtain the upper level for the predictive accuracy of the selected consensus proteins, the final model was fit to the full dataset and the predictive accuracy was quantified using the area under the ROC curve, sensitivity, specificity, and accuracy. Standard errors of these characteristics were derived from 2,000 bootstrap replicates. Moreover, an unbiased estimate of the predictive ability of the selected proteins was obtained by the pseudomedian fold of the cross-validation step, which corresponds to the 5^th^ largest AUC value out of the ten folds. Finally, an estimate of variability associated with the ROC curve was obtained by plotting the 25^th^ and the 75^th^ quantile of the sensitivities for each value of 1-specificity over ten folds.

To evaluate the stability of the final models, eight-fold cross-validation was used. Applying the identical methodology to the 10-fold procedure (except that the final consensus model for each endpoint comprised proteins selected more than four times among the eight folds), the obtained 8-fold final models consisted of similar protein signatures and performances showed similar properties to the 10-fold ones. Likelihood-ratio test was applied to compare the consensus Cox model and Cox model with clinical factors only. To determine the added value of the consensus model (i.e. outcome biomarker signature), survival curves were visualized in two ways. First, the Cox model was fitted with stage alone or with stage and the linear protein combination, and survival was predicted. Age and gender were kept fixed. Second, survival was stratified by stage alone or with stage and the linear protein combination, and the survival curves were drawn for the respected groups of subjects. Age and gender were unaccounted for. Pseudocode of predictive analyses is available in the Appendix. The pROC and survivalROC packages in R were used to draw ROCs and to calculate AUCs and other performances (i.e. sensitivity, specificity, and accuracy). For bootstrap analysis, the boot package was used. The survival R package was used to perform the Cox analyses and survival comparisons.

### Validation with external proteomic and transcriptomic datasets

All published data were used as originally normalized and transformed by the authors.

### Functional analysis of signature proteins

Ingenuity Pathway Analysis (version 18488943, www.ingenuity.com) was employed to associate disease annotations stored in the Ingenuity Knowledge Base to the signature proteins. A “functional analysis” was used to identify the disease categories associated with the proteins. The association significance was calculated with the right-tailed Fisher’s exact test. Results were filtered under the “disease & functions” tab for the high-level (i.e. primary) category: cancer. Low-level (i.e. secondary) categories “cancer” and “colorectal cancer” were used to view the associated genes. Proteins were further annotated with gene ontology (GO) biological process terms. Protein accessions were loaded in Panther 9.0 Classification System where “Functional classification viewed in gene list” analysis was performed. GO biological process terms were further grouped into four major categories and proteins were linked to their category graphically in Cytoscape v3.0.2 to illustrate the overlap between biological terms.

## For more information

UniProt, the Universal Protein Resource for protein sequence and annotation data. http://www.uniprot.org/

SRMAtlas, a compendium of targeted proteomics assays for the detection and quantification of proteins in complex proteome digests by mass spectrometry.

http://www.srmatlas.org/

PASSEL, the PeptideAtlas SRM Experiment Library for the submission, dissemination, and reuse of SRM experimental data. http://www.peptideatlas.org/passel/

MSstats, a statistical tool for quantitative mass spectrometry-based proteomics employing a family of linear mixed-effects models. http://www.msstats.org/

Gene Expression Omnibus, a genomics data repository of array- and sequence-based data. http://www.ncbi.nlm.nih.gov/geo/

## Supporting Information

**Appendix**

**Review Process File**
