# Supplementary material for: Non-invasive prognostic protein biomarker signatures associated with colorectal cancer
Source: EMBO Mol Med. 2015 Aug 7;7(9):1153–65. doi: 10.15252/emmm.201404874 (PMC4568949; doi:10.15252/emmm.201404874)
Supplement: Supplementary file 1 [file emmm0007-1153-sd1.pdf]

Appendix

Non-invasive prognostic protein biomarker signatures associated with colorectal cancer

Silvia Surinova, Lenka Radová, Meena Choi, Josef Srovnal, Hermann Brenner, Olga Vitek, Marián Hajdúch, Ruedi Aebersold

Table of contents

Appendix information

Pseudocode of predictive analyses

Appendix figures

Appendix Fig. S1. Stratification of survival based on the biomarker signature of CRC outcome

Appendix Fig. S2. Evaluation of the outcome biomarker signature in the GSE17536 transcriptomic data associated with TNM staging and overall survival (OS)

Appendix Fig. S3. Evaluation of the outcome biomarker signature in the GSE14333 transcriptomic data associated with Dukes staging and disease-free survival (DFS)

Appendix Fig. S4. Evaluation of the localization biomarker signature in proteomic and transcriptomic data sets acquired from the TCGA cohort

Appendix Fig. S5. Evaluation of the dissemination biomarker signature in the transcriptomic data set acquired from the TCGA cohort

Appendix tables

Appendix Table S1. Detectable candidate proteins in patient plasma

Appendix Table S2. Outcome biomarker signature development within 10-fold CV

Appendix Table S3. Reproducibility assessment of the outcome biomarker signature within 8-fold CV

Appendix Table S4. Regional localization biomarker signature development within 10-fold CV

Appendix Table S5. Reproducibility assessment of the regional localization biomarker signature within 8-fold CV

Appendix Table S6. Grading biomarker signature development within 10-fold CV

Appendix Table S7. Clinical stage biomarker signature development within 10-fold CV

Appendix Table S8. Disseminated disease biomarker signature development within 10-fold CV

Appendix Table S9. Reproducibility assessment of the disseminated disease biomarker signature within 8-fold CV

Appendix Table S10. Forced selection of clinical factors (i.e. age, gender, and stage) into predictive biomarker signatures for **a**, regional localization, and **b**, TNM metastasis status

Appendix Table S11. The performance of individual outcome signature proteins on the protein or transcript levels

Appendix Table S12. Classification of colon cancer subtypes (CCSs) based on the outcome signature proteins within 10-fold cross-validation of the GSE33113 data set.

Appendix Table S13. Classification of the five cellular phenotype subtypes based on the outcome signature proteins within **a**, 5-fold cross-validation of the GSE13294 data set, and **b**, 10-fold cross-validation of the GSE14333 data set.

Appendix Table S14. The performance of individual localization signature proteins on the protein or transcript levels

Appendix Table S15. Functional annotation of signature proteins with gene ontology (GO) biological process terms

Additional information

Pseudocode of predictive analyses

5year overall survival – COX MODEL

1. Dataset = patients in stages 1+2+3
2. denote: 0 = patient died until 5years, 1 = patient censored until 5years, 2 = patient survived 5 years timepoint
3. split patients into 10 folds with equivalent proportions of died until 5years (0), censored during 5years (1) and alive in 5years timepoint patients as in the whole dataset.
4. repeat for i in 1:10

a) PROT_i = Preselected proteins by SRMstats

b) VALIDATION_i = patients in fold i

c) TRAINING_i = patients in all remaining folds (except i-th fold)

d) MODEL_i = results of stepwise selection for Cox model with forced predictors (age, gender, stage) applied on dataset TRAINING_i, with variables PROT_i (model with minimal Akaike information criteria)

e) ROC_i = survivalROC analysis for MODEL_i on dataset VALIDATION_i in timepoint t=5years

f) SIGNPROT_i = proteins from MODEL_i

5. PROT_FINAL = proteins, which occur at least 5-times in SIGNPROT_1, …, SIGNPROT_10
6. FULL_MODEL = Cox model on all patients with variables age, gender, stage and PROT_FINAL
7. survivalROC analysis on FULL_MODEL in timepoint t=5years; output: black ROC curve in fig. 2a
8. bootstrap for censored data applied on FULL_MODEL with 2000 replicate; output in table: standard errors of AUC, sensitivity, specificity and accuracy for FULL_MODEL
9. ROC_med = ROC_i with 5th maximal AUC among ROC_1, …, ROC_10 in timepoint t=5years
10. plot ROC_med; output: red ROC curve in fig. 2a
11. inter-quartile range of ROC_1, …, ROC_10 in each point; output: grey area in fig 2a
12. AUC, sensitivity, specificity and accuracy for ROC_1, … ,ROC_10 in timepoint t=5years; output in table: standard errors of AUC, sensitivity, specificity and accuracy
13. CLIN_MODEL = Cox model on all patients with variables age, gender, stage
14. LR test to compare FULL_MODEL and CLIN_MODEL; output: p-value in text
15. PRED_STAGE = individual predictions of CLIN_MODEL for patients in dataset with fixed age:: 68 and fixed gender::MALE
16. plot PRED_STAGE; output fig. 2b
17. PROT_MODEL = Cox model with predictors PROT_FINAL
18. PRED_PROT = individual predictions of PROT_MODEL

19. CATEGORY – split patients into 2 subsets

a) “HIGHrisk” – patients with PRED_PROT = median(PRED_PROT)

b) “LOWrisk” – patients with PRED_PROT < median(PRED_PROT)

20. MIXED_MODEL = Cox model with predictors age, gender, stage, CATEGORY

21. plot individual predictions of MIXED_MODEL with fixed age::68, gender::MALE and stage::1 (output fig.2c), stage::2 (output fig. 2d), stage::3 (output fig. 2e)

22. survival plot for stage; output Appendix fig. S1a

23. survival plot for CATEGORY with patients in stage 1; output: Appendix fig. S1b)

survival plot for CATEGORY with patients in stage 2; output: Appendix fig. S1c)

survival plot for CATEGORY with patients in stage 3; output: Appendix fig. S1d)

Regional localization (CRC colon vs CRC rectum) – LOGISTIC MODEL

1. denote: 0 = patient with DG colon, 1 = patient with DG rectum

2. split patients into 10 folds with equivalent proportions of diagnosis colon (0) and rectum (1) as in the whole dataset.

3. repeat for i in 1:10

a) PROT_i = Preselected proteins by SRMstats

b) VALIDATION_i = patients in fold i

c) TRAINING_i = patients in all remaining folds (except i-th fold)

d) MODEL_i = results of stepwise selection with logistic model applied on dataset TRAINING_i, with variables PROT_i (model with minimal Akaike information criteria)

e) ROC_i = ROC analysis for MODEL_i on dataset VALIDATION_i

f) SIGNPROT_i = proteins from MODEL_i

4. PROT_FINAL = proteins, which occur at least 5-times in SIGNPROT_1, …, SIGNPROT_10

5. FULL_MODEL = logistic regression model on all patients with variables PROT_FINAL

6. ROC analysis on FULL_MODEL; output: black ROC curve in fig. 4a

7. bootstrap FULL_MODEL with 2000 replicate; output in table: standard errors of AUC, sensitivity, specificity and accuracy for FULL_MODEL

8. ROC_med = ROC_i with 5th maximal AUC among ROC_1, …, ROC_10

9. plot ROC_med; output: red ROC curve in fig. 4a

10. inter-quartile range of ROC_1, …, ROC_10 in each point; output: grey area in fig. 4a

11. AUC, sensitivity, specificity and accuracy for ROC_1, … ,ROC_10; output in table: standard errors of AUC, sensitivity, specificity and accuracy

TNM metastasis status (CRC stage 1+2+3 vs CRC stage 4) – LOGISTIC MODEL

1. denote: 0 = patient in stages 1+2+3, 1 = patient in stage4
2. split patients into 10 folds with equivalent proportions of early stages (0) and developed illness (1) as in the whole dataset.
3. repeat for i in 1:10

a) PROT_i = Preselected proteins by SRMstats

b) VALIDATION_i = patients in fold i

c) TRAINING_i = patients in all remaining folds (except i-th fold)

d) MODEL_i = results of stepwise selection with logistic model applied on dataset TRAINING_i, with variables PROT_i (model with minimal Akaike information criteria)

e) ROC_i = ROC analysis for MODEL_i on dataset VALIDATION_i

f) SIGNPROT_i = proteins from MODEL_i

4. PROT_FINAL = proteins, which occur at least 5-times in SIGNPROT_1, …, SIGNPROT_10
5. FULL_MODEL = logistic regression model on all patients with variables PROT_FINAL
6. ROC analysis on FULL_MODEL; output: black ROC curve in fig. 4b
7. bootstrap FULL_MODEL with 2000 replicate; output in table: standard errors of AUC, sensitivity, specificity and accuracy for FULL_MODEL
8. ROC_med = ROC_i with 5th maximal AUC among ROC_1, …, ROC_10
9. plot ROC_med; output: red ROC curve in fig. 4b
10. inter-quartile range of ROC_1, …, ROC_10 in each point; output: grey area in fig. 4b
11. AUC, sensitivity, specificity and accuracy for ROC_1, … ,ROC_10; output in table: standard errors of AUC, sensitivity, specificity and accuracy

Appendix figures

Appendix Fig. S1. Stratification of survival based on the biomarker signature of CRC outcome. All collected survival data was used to plot stratified-survival based on a) stage 1, 2, or 3; b) stage 1 and signature proteins; c) stage 2 and signature proteins; and d) stage 3 and signature proteins. The signature proteins represent a linear combination of protein intensities (0.739*HLA-A - 1.143*CFH + 0.811*CD44 + 0.334*PTPRJ + 0.398*HP - 0.869*CDH5). The cutoff of -0.03685376 used for stratification is the median of individual predictions for all patients in stages 1+2+3. HIGH risk group represents patients with individual predictions≥cutoff and LOW risk group represents patients with individual predictions<cutoff. In the HIGH risk group, n=17 are at stage 1, n=29 are at stage 2, n=29 are at stage 3. In the LOW risk group, n=23 are at stage 1, n=29 are at stage 2, and n=22 are at stage 3. n represents the number of patients in each category.

Appendix Fig. S2. Evaluation of the outcome biomarker signature in the GSE17536 transcriptomic data associated with TNM staging and overall survival (OS). Data from 138 patients of stage I-III were used in this analysis, where 35 patients died until 5 years, 54 patients were censored until 5 years, and 49 patients survived longer than 5 years. a) Biomarker signature containing clinical factors (age, gender, stage) and gene proxies of all signature proteins predicting 5-year overall survival within 5-fold cross validation. The performance obtained on the cross-validated pseudomedian validation fold (i.e. between fold median; labeled in red), corresponding 25th (in magenta) and 75th (in orange) percentile bounds, and on the full data set (labeled in black). The individual training and validation areas under the ROC curves from the 5-fold cross validation are reported in the adjacent table. b-e) All collected survival data was used to plot predicted survival based on the Cox model fitted with fixed parameters (age=67, gender=male) and with b) stage 1, 2, or 3; c) stage 1 and signature genes; d) stage 2 and signature genes; and e) stage 3 and signature genes. HIGH risk group represents patients with individual predictions≥cutoff and LOW risk group represents patients with individual predictions<cutoff.

Appendix Fig. S3. Evaluation of the outcome biomarker signature in the GSE14333 transcriptomic data associated with Dukes staging and disease-free survival (DFS). Data from 139 patients of Dukes stage A-C were used in this analysis, where 21 patients relapsed until 5 years, 88 patients were censored until 5 years, and 30 patients survived disease-free longer than 5 years. a) Biomarker signature containing clinical factors (age, gender, stage) and gene proxies of all signature proteins predicting 5-year DFS within 10-fold cross validation. The performance obtained on the cross-validated pseudomedian validation fold (i.e. between fold median; labeled in red), corresponding 25th (in magenta) and 75th (in orange) percentile bounds, and on the full data set (labeled in black). The individual training and validation areas under the ROC curves from the 10-fold cross validation are reported in the adjacent table. b-e) All collected survival data was used to plot predicted survival based on the Cox model fitted with fixed parameters (age=71, gender=male) and with b) stage A, B, or C; c) stage A and signature genes; d) stage B and signature genes; and e) stage C and signature genes. HIGH risk group represents patients with individual predictions≥cutoff and LOW risk group represents patients with individual predictions<cutoff.

Appendix Fig. S4. Evaluation of the localization biomarker signature in proteomic and transcriptomic data sets acquired from the TCGA cohort. The performance of the signature proteins was assessed in a) 88 patients (colon tumors: n=58, rectal tumors: n=30) on the protein level, b) the same 88 patients on the transcript level, and c) a larger set of 270 patients (colon tumors: n=196, rectal tumors: n=74) on the transcript level.

Appendix Fig. S5. Evaluation of the dissemination biomarker signature in the transcriptomic data set acquired from the TCGA cohort. The performance of the signature proteins was assessed in the full set of 270 patients (localized CRC: n=224, metastatic CRC: n=40) on the transcript level.

Appendix tables

Appendix Table S1. Detectable candidate proteins in patient plasma. Gene names, protein names, and accession codes were defined according to UniProt Knowledgebase (www.uniprot.org).

Gene name	Accession	Protein name
A1AG2	P19652	Alpha-1-acid glycoprotein 2
AFM	P43652	Afamin
AHSG	P02765	Alpha-2-HS-glycoprotein (Fetuin-A)
ANPEP	P15144	Aminopeptidase N
ANT3	P01008	Antithrombin-III (Serpin C1)
AOC3	Q16853	Membrane primary amine oxidase
APMAP	Q9HDC9	Adipocyte plasma membrane-associated protein
APOB	P04114	Apolipoprotein B-100
ATRN	O75882	Attractin
B3GN2	Q9NY97	UDP-GlcNAc:betaGal beta-1,3-N-acetylglucosaminyltransferase 2
BTD	P43251	Biotinidase (Biotinase)
CADM1	Q9BY67	Cell adhesion molecule 1
CD109	Q6YHK3	CD109 antigen
CD163	Q86VB7	Scavenger receptor cysteine-rich type 1 protein M130
CD44	P16070	CD44 antigen (Extracellular matrix receptor III) (Hyaluronate receptor)
CDH5	P33151	Cadherin-5 (CD144)
CFH	P08603	Complement factor H
CFI	P05156	Complement factor I
CLU	P10909	Clusterin
CNTN4	Q8IWV2	Contactin-4
CO4A	P0C0L4	Complement C4-A
CP	P00450	Ceruloplasmin
CTSD	P07339	Cathepsin D
DKFZp686C02220	Q6N091	Putative uncharacterized protein DKFZp686C02220
DPEP1	P16444	Dipeptidase 1
DSG2	Q14126	Desmoglein-2 (Cadherin family member 5)
ECM1	Q16610	Extracellular matrix protein 1
F11	P03951	Coagulation factor XI
F5	P12259	Coagulation factor V
FCGBP	Q9Y6R7	IgGFc-binding protein
FETUB	Q9UGM5	Fetuin-B
FGA	P02671	Fibrinogen alpha chain
FGG	P02679	Fibrinogen gamma chain
FHR3	Q02985	Complement factor H-related protein 3
FN1	P02751	Fibronectin
GOLM1	Q8NBJ4	Golgi membrane protein 1 (Golgi phosphoprotein 2)
HLA-A	P01892	HLA class I histocompatibility antigen, A-2 alpha chain
HP	P00738	Haptoglobin
HPX	P02790	Hemopexin (Beta-1B-glycoprotein)
HRG	P04196	Histidine-rich glycoprotein
HYOU1	Q9Y4L1	Hypoxia up-regulated protein 1

ICAM1	P05362	Intercellular adhesion molecule 1 (CD54)
ICAM2	P13598	Intercellular adhesion molecule 2 (CD102)
IGFBP3	P17936	Insulin-like growth factor-binding protein 3
IGHA2	P01877	Ig alpha-2 chain C region
IGHG1	P01857	Ig gamma-1 chain C region
IGHG2	P01859	Ig gamma-2 chain C region
IGHM	P01871	Ig mu chain C region
IGJ	P01591	Immunoglobulin J chain
ISLR	O14498	Immunoglobulin superfamily containing leucine-rich repeat protein
ITIH4	Q14624	Inter-alpha-trypsin inhibitor heavy chain H4
KDR	P35968	Vascular endothelial growth factor receptor 2 (CD309)
KLKB1	P03952	Plasma kallikrein (Kininogenin)
KNG1	P01042	Kininogen-1 (Alpha-2-thiol proteinase inhibitor)
LAMA2	P24043	Laminin subunit alpha-2
LAMP2	P13473	Lysosome-associated membrane glycoprotein 2 (CD107b)
LCN2	P80188	Neutrophil gelatinase-associated lipocalin (Oncogene 24p3)
LGALS3BP	Q08380	Galectin-3-binding protein (Mac-2 BP) (Tumor-associated antigen 90K)
LRG1	P02750	Leucine-rich alpha-2-glycoprotein
LUM	P51884	Lumican
LYVE1	Q9Y5Y7	Lymphatic vessel endothelial hyaluronic acid receptor 1
MFAP4	P55083	Microfibril-associated glycoprotein 4
MMRN1	Q13201	Multimerin-1 (EMILIN-4)
MPO	P05164	Myeloperoxidase
MRC2	Q9UBG0	C-type mannose receptor 2 (CD280)
MST1	Q13043	Serine/threonine-protein kinase 4
NCAM1	P13591	Neural cell adhesion molecule 1 (CD56)
NEO1	Q92859	Neogenin
ORM1	P02763	Alpha-1-acid glycoprotein 1
PGCP	Q9Y646	Plasma glutamate carboxypeptidase
PIGR	P01833	Polymeric immunoglobulin receptor (Hepatocellular carcinoma-associated protein TB6)
PLTP	P55058	Phospholipid transfer protein
PLXDC2	Q6UX71	Plexin domain-containing protein 2 (Tumor endothelial marker 7-related protein)
PLXNB2	O15031	Plexin-B2
PON1	P27169	Serum paraoxonase/arylesterase 1
PRG4	Q92954	Proteoglycan 4
PROC	P04070	Vitamin K-dependent protein C
PTPRJ	Q12913	Receptor-type tyrosine-protein phosphatase eta (CD148)
SERPINA1	P01009	Alpha-1-antitrypsin (Serpin A1)
SERPINA3	P01011	Alpha-1-antichymotrypsin (Cell growth-inhibiting gene 24/25 protein) (Serpin A3)
SERPINA6	P08185	Corticosteroid-binding globulin (Serpin A6)
SERPINA7	P05543	Thyroxine-binding globulin (Serpin A7)
THBS1	P07996	Thrombospondin-1
TIMP1	P01033	Tissue inhibitor of metalloproteinases 1
TNC	P24821	Tenascin (Tenascin-C)
TRF	P02787	Serotransferrin (Transferrin)
VTN	P04004	Vitronectin
VWF	P04275	von Willebrand factor

Appendix Table S2. Outcome biomarker signature development within 10-fold CV. **a**, Differentially abundant proteins characterised as significant in the individual folds of the training dataset. **b**, Proteins selected into Cox regression models in individual folds. The consensus model contains clinical factors and proteins with a high frequency of occurrence in the individual folds. AUC values are reported. The median AUC presented in figure 2 is in bold. **c**, Parameters of the consensus Cox model, the linear combination of proteins, and the selected cutoff for survival prediction.

**a**	**Significant proteins for each fold (FDR<0.05, fold change cut-off ±1.1)**
Fold	Differentially abundant proteins
1	A1AG2, AFM, ANT3, APMAP, CD109, CD44, CDH5, CFH, CP, CTSD, DKFZp686N02209, DSG2, ECM1, F11, FETUB, FGA, FGG, HLA-A, HP, HYOU1, IGJ, ITIH4, KNG1, LAMP2, LCN2, LGALS3BP, LUM, MFAP4, MMRN1, MPO, MRC2, NCAM1, ORM1, PIGR, PTPRJ, SERPINA6, SERPINA7, TIMP1
2	ANT3, APMAP, CD109, CD44, CDH5, CFH, CP, CTSD, FETUB, FGA, FGG, HLA-A, HP, HRG, IGJ, ITIH4, KNG1, LAMP2, LCN2, LGALS3BP, LUM, LYVE1, MFAP4, MMRN1, MRC2, MST1, ORM1, PIGR, PLXDC2, PTPRJ, SERPINA6, SERPINA7, TIMP1, VTN
3	A1AG2, ANT3, APMAP, CD109, CD44, CDH5, CFH, CP, CTSD, ECM1, FCGBP, FETUB, FGA, FGG, HLA-A, HP, IGJ, ITIH4, KNG1, LAMP2, LGALS3BP, LRG1, MMRN1, MPO, MRC2, MST1, NCAM1, ORM1, PIGR, PTPRJ, SERPINA7, TIMP1, TNC, VTN
4	A1AG2, ANT3, APOB, APMAP, CD109, CD44, CDH5, CFH, CP, CTSD, DSG2, FCGBP, FETUB, FGA, FGG, HLA-A, HP, IGJ, ITIH4, KLKB1, KNG1, LAMP2, LGALS3BP, LRG1, LUM, MMRN1, MPO, MRC2, MST1, ORM1, PIGR, PTPRJ, SERPINA3, SERPINA6, SERPINA7, TIMP1, VTN
5	A1AG2, ANT3, CADM1, CD109, CD44, CFH, CP, CTSD, DSG2, ECM1, FCGBP, FETUB, FGA, FGG, HLA-A, HP, HYOU1, ICAM1, IGHM, IGJ, ITIH4, KLKB1, KNG1, LAMP2, LGALS3BP, LUM, MMRN1, MPO, MRC2, MST1, ORM1, PIGR, PLXDC2, PTPRJ, TIMP1, VTN
6	A1AG2, AFM, ANT3, APMAP, CD109, CD44, CDH5, CFH, CP, CTSD, DKFZp686N02209, DSG2, ECM1, F5, FCGBP, FETUB, FGA, FGG, HLA-A, HP, HYOU1, IGJ, KNG1, LAMP2, LCN2, LGALS3BP, LUM, MMRN1, MPO, MRC2, MST1, NCAM1, ORM1, PIGR, PLXDC2, PTPRJ, TIMP1, TNC
7	ANT3, CD109, CD44, CDH5, CFH, CP, F11, FCGBP, FETUB, FGA, FGG, HLA-A, HP, IGJ, LGALS3BP, MMRN1, MPO, MRC2, PIGR, PLXDC2, PTPRJ, TIMP1
8	A1AG2, ANT3, APMAP, CD44, CDH5, CFH, CP, DKFZp686N02209, ECM1, FETUB, FGA, FGG, GOLM1, HLA-A, HP, HYOU1, IGJ, KNG1, LGALS3BP, LUM, MMRN1, MPO, MRC2, NCAM1, ORM1, PIGR, PLXDC2, PTPRJ, SERPINA6, TIMP1, VTN
9	ANT3, APMAP, CD109, CD44, CDH5, CFH, CP, CTSD, FCGBP, FGA, FGG, HLA-A, HP, LAMP2, LGALS3BP, MFAP4, MMRN1, MPO, MRC2, MST1, ORM1, PIGR, PLXDC2, PTPRJ, SERPINA3, SERPINA7, TIMP1, VTN
10	ANT3, APMAP, CD109, CD44, CDH5, CFH, CP, CTSD, DSG2, F11, FGA, FGG, HLA-A, HP, IGJ, ITIH4, KNG1, LAMP2, LGALS3BP, MFAP4, MMRN1, MPO, MRC2, MST1, NCAM1, ORM1, PGCP, PTPRJ, Q5JNX2, TIMP1, VTN

**b**	**Significant proteins selected into Cox regression models by stepwise selection**		
Fold	Predictive Cox regression models	Training (9/10 dataset)	Validation (1/10 dataset)
1	Gender, Age, Stage, CD44, CDH5, CFH, DKFZp686N02209, DSG2, FETUB, HLA-A, HP, IGJ, PTPRJ	0.77	0.91
2	Gender, Age, Stage, CDH5, CFH, FETUB, HLA-A, HP, PTPRJ	0.73	0.70
3	Gender, Age, Stage, A1AG2, CD44, CDH5, CFH, FCGBP, HLA-A, ITIH4	0.74	0.88
4	Gender, Age, Stage, HLA-A, HP, KNG1, LAMP2	0.71	0.79
5	Gender, Age, Stage, DSG2, FCGBP, FGA, HLA-A, KNG1, LAMP2, PTPRJ	0.76	**0.75**
6	Gender, Age, Stage, AFM, CDH5, DKFZp686N02209, DSG2, FCGBP, HLA-A, PTPRJ, TNC	0.73	0.84
7	Gender, Age, Stage, CD44, CDH5, CFH, FCGBP, FGG, HLA-A	0.73	0.74
8	Gender, Age, Stage, CD44, CFH, FETUB, GOLM1, HLA-A, HP, HYOU1, IGJ, LUM, MRC2, PTPRJ, SERPINA6	0.77	0.73

9	Gender, Age, Stage, CD44, CFH, HP, MPO, PTPRJ	0.74	0.74
10	Gender, Age, Stage, CD109, CD44, CFH, DSG2, HLA-A	0.73	0.70
Consensus	Gender, Age, Stage, HLA-A, CFH, CD44, PTPRJ, HP, CDH5	0.72	
**c**	**Consensus Cox model**		
Model	0.01524931*Age - 0.07394655*Gender(male=1, female=0) + 0.40446192*Stage + 0.77439420*HLA-A - 0.91423495*CFH + 0.59998490*CD44 + 0.36973892*PTPRJ + 0.38526529*HP - 0.77461635*CDH5		
Cutoff	-0.001		

Appendix Table S3. Reproducibility assessment of the outcome biomarker signature within 8-fold CV. **a**, Differentially abundant proteins characterised as significant in the individual folds of the training dataset. **b**, Proteins selected into Cox regression models in individual folds. The consensus model contains clinical factors and proteins with a high frequency of occurrence in the individual folds. AUC values are reported.

**a**	**Significant proteins for each fold (FDR<0.05, fold change cut-off ±1.1)**		
Fold	Differentially abundant proteins		
1	ANT3, APMAP, CADM1, CD109, CD44, CFH, CP, CTSD, DKFZp686N02209, DSG2, FETUB, FGA, FGG, HLA-A, HP, HYOU1, IGHA2, IGJ, LAMP2, LGALS3BP, MFAP4, MMRN1, MPO, MRC2, MST1, NCAM1, ORM1, PIGR, PLXDC2, PTPRJ, TIMP1		
2	A1AG2, ANT3, APMAP, CADM1, CD44, CDH5, CP, FGA, FGG, HLA-A, HP, HYOU1, IGJ, ITIH4, KNG1, LGALS3BP, MFAP4, MMRN1, MRC2, MST1, NCAM1, ORM1, PIGR, PTPRJ, SERPINA6, SERPINA7, TIMP1, VWF		
3	A1AG2, AFM, ANT3, APMAP, CADM1, CD109, CD44, CDH5, CFH, CP, CTSD, DKFZp686N02209, ECM1, F11, FGA, FGG, HLA-A, HP, HYOU1, ICAM1, IGJ, ITIH4, KNG1, LAMP2, LGALS3BP, LRG1, MFAP4, MMRN1, MPO, MRC2, MST1, NCAM1, ORM1, PIGR, PTPRJ, SERPINA6, SERPINA7, TIMP1		
4	A1AG2, AFM, ANT3, APOB, APMAP, CADM1, CD109, CD44, CDH5, CFH, CP, CTSD, DSG2, FETUB, FGA, FGG, HLA-A, HP, ICAM1, IGJ, ITIH4, KNG1, LAMP2, LGALS3BP, LRG1, LUM, MFAP4, MMRN1, MPO, MRC2, MST1, ORM1, PGCP, PIGR, PLXDC2, PTPRJ, Q5JNX2, SERPINA3, SERPINA6, SERPINA7, TIMP1, VTN		
5	ANT3, CD109, CD44, CDH5, CFH, FCGBP, FETUB, FGA, FGG, HLA-A, HP, LAMP2, LGALS3BP, MMRN1, MPO, MRC2, MST1, PIGR, PLXDC2, PTPRJ, TIMP1, VTN		
6	A1AG2, AFM, ANT3, APOB, APMAP, CADM1, CD109, CD44, CDH5, CFH, CP, CTSD, DKFZp686N02209, DSG2, ECM1, FETUB, FGA, FGG, HLA-A, HP, HYOU1, ICAM1, IGJ, ITIH4, KNG1, LAMP2, LGALS3BP, MFAP4, MMRN1, MPO, MRC2, MST1, NCAM1, ORM1, PLXDC2, PTPRJ, SERPINA7, TIMP1, VTN		
7	ANT3, CD109, CD44, CDH5, CFH, CP, FETUB, FGA, FGG, HLA-A, IGJ, ITIH4, MPO, MRC2, MST1, PIGR, SERPINA6		
8	ANT3, APMAP, CADM1, CD109, CD44, CDH5, CFH, FETUB, FGA, FGG, FN1, HLA-A, HP, KNG1, LAMP2, LUM, MFAP4, MMRN1, MPO, MRC2, NCAM1, PIGR, PTPRJ, VTN		
**b**	**Significant proteins selected into Cox regression models by stepwise selection**		
Fold	Predictive Cox regression models	Training (7/8 dataset)	Validation (1/8 dataset)
1	Gender, Age, Stage, CADM1, HP, PTPRJ	0.71	0.74
2	Gender, Age, Stage, A1AG2, APMAP, HLA-A, PTPRJ, SERPINA6, VWF	0.76	0.69
3	Gender, Age, Stage, CD44, CDH5, CFH, HLA-A, HP, ICAM1, PTPRJ	0.75	0.76
4	Gender, Age, Stage	0.63	0.52
5	Gender, Age, Stage, CD44, CDH5, CFH, HLA-A, LAMP2	0.73	0.80
6	Gender, Age, Stage, AFM, DKFZp686N02209, FETUB, HLA-A, HYOU1, PTPRJ	0.74	0.77
7	Gender, Age, Stage, CD44, CDH5, CFH, FETUB, HLA-A	0.73	0.66
8	Gender, Age, Stage, APMAP, CADM1, CD109,	0.78	0.86

	CD44, CDH5, CFH, HLA-A, LUM, MFAP4, MRC2, PIGR, PTPRJ		
Consensus	Gender, Age, Stage, CD44, CDH5, CFH, PTPRJ, HLA-A		0.71

Appendix Table S4. Regional localization (colon versus rectum) biomarker signature development within 10-fold CV. **a**, Differentially abundant proteins characterised as significant in the individual folds of the training dataset. **b**, Proteins selected into logistic regression models in individual folds. The consensus model contains proteins with a high frequency of occurrence in the individual folds. AUC values are reported. The median AUC presented in figure 2 is in bold. **c**, Parameters of the consensus model, the linear combination of proteins, and the selected cutoff for regional localization prediction.

**a**	**Significant proteins for each fold (FDR<0.05, fold change cut-off ±1.1)**		
Fold	Differentially abundant proteins		
1	CADM1, CD163, CDH5, CTSD, F5, FHR3, FN1, GOLM1, HLA-A, HRG, HYOU1, KDR, LCN2, LGALS3BP, LRG1, MRC2, ORM1, PON1, TIMP1, VTN		
2	CADM1, CD163, CD44, CDH5, CFH, CP, CTSD, F5, FHR3, FN1, GOLM1, HLA-A, HYOU1, LCN2, LGALS3BP, LRG1, MFAP4, MRC2, ORM1, PON1, PRG4, TIMP1, VTN		
3	CADM1, CD163, CDH5, CP, CTSD, F5, FETUB, FHR3, FN1, GOLM1, HLA-A, HYOU1, ICAM1, ICAM2, IGHM, LCN2, LGALS3BP, LRG1, MFAP4, MRC2, ORM1, PON1, PRG4, TIMP1, VTN		
4	CADM1, CD109, CD163, CDH5, CP, CTSD, F5, FHR3, GOLM1, HLA-A, HYOU1, LCN2, LGALS3BP, LRG1, MPO, MRC2, ORM1, PON1, TIMP1, VTN		
5	CADM1, CD109, CD163, CDH5, CFH, CP, CTSD, F5, FCGBP, FHR3, HLA-A, HYOU1, KDR, LGALS3BP, LRG1, MFAP4, MPO, MRC2, ORM1, PRG4, TIMP1		
6	CADM1, CD163, CFH, CTSD, F5, FCGBP, FHR3, FN1, HLA-A, HYOU1, LGALS3BP, LRG1, MPO, MRC2, ORM1, PIGR, PON1, PRG4, Q6N091, TIMP1, VTN		
7	CADM1, CD109, CD163, CTSD, F5, FHR3, FN1, HLA-A, HYOU1, LCN2, LGALS3BP, LRG1, MPO, MRC2, ORM1, PON1, TIMP1, VTN		
8	CADM1, CD163, CP, CTSD, F5, FHR3, FN1, GOLM1, HLA-A, HYOU1, LCN2, LGALS3BP, LRG1, MPO, MRC2, ORM1, PON1, TIMP1, VTN		
9	CADM1, CD163, CD44, CDH5, CP, CTSD, F5, FHR3, FN1, HLA-A, HP, HYOU1, LCN2, LGALS3BP, LRG1, MFAP4, MMRN1, MPO, MRC2, ORM1, PON1, PRG4, TIMP1, VTN		
10	A1AG2, APMAP, CADM1, CD163, CD44, CDH5, CFH, CP, CTSD, F5, FHR3, FN1, GOLM1, HLA-A, HYOU1, ICAM1, ICAM2, LCN2, LGALS3BP, LRG1, MPO, MRC2, ORM1, PRG4, Q6N091, SERPINA1, TIMP1, TNC		
**b**	**Significant proteins selected into logistic regression models by stepwise selection**		
Fold	Predictive logistic regression models	Training (9/10 dataset)	Validation (1/10 dataset)
1	CADM1, FN1, HRG, HYOU1, LGALS3BP, LRG1, MRC2, TIMP1, VTN	0.79	0.48
2	CADM1, FN1, HYOU1, LGALS3BP, MRC2, VTN	0.73	0.82
3	CADM1, FN1, HYOU1, LGALS3BP, VTN	0.74	0.58
4	CADM1, CD109, LGALS3BP, LRG1, MRC2, PON1	0.76	0.56
5	CADM1, CD109, HYOU1, LGALS3BP, LRG1	0.75	**0.66**
6	CADM1, FN1, HYOU1, LGALS3BP, MRC2, VTN	0.76	0.43
7	CADM1, CD109, FN1, HYOU1, LGALS3BP, LRG1, MRC2, VTN	0.77	0.71
8	CADM1, FN1, HYOU1, LGALS3BP, LRG1, MRC2, VTN	0.74	0.81
9	CADM1, FN1, HP, HYOU1, LGALS3BP, LRG1, MMRN1, VTN	0.77	0.80
10	CADM1, FN1, HYOU1, ICAM2, LGALS3BP	0.75	0.53
Consensus	CADM1, LGALS3BP, HYOU1, FN1, VTN, LRG1, MRC2		0.75
**c**	**Consensus model** where rectal tumors=1, others =0		
Model	15.6909187 - 0.5859094*CADM - 0.5426449*LGALS3BP - 0.5356852*HYOU1 + 0.4039757*FN1		

	+ 0.4549288*VTN - 0.3022558*LRG1 - 0.7189315*MRC2
Cutoff	0.272

Appendix Table S5. Reproducibility assessment of the regional localization (colon versus rectum) biomarker signature within 8-fold CV. **a**, Differentially abundant proteins characterised as significant in the individual folds of the training dataset. **b**, Proteins selected into logistic regression models in individual folds. The consensus model contains proteins with a high frequency of occurrence in the individual folds. AUC values are reported.

**a**	**Significant proteins for each fold (FDR<0.05, fold change cut-off ±1.1)**
Fold	Differentially abundant proteins
1	ATRN, CADM1, CD109, CD163, CD44, CDH5, CFH, CLU, CTSD, F5, FHR3, FN1, HLA-A, HYOU1, IGHG2, IGHM, LCN2, LGALS3BP, LRG1, LUM, MPO, MRC2, ORM1, PON1, PRG4, VTN
2	A1AG2, CADM1, CD109, CD163, CDH5, CFH, CTSD, F5, FHR3, HLA-A, HYOU1, ICAM1, IGJ, LCN2, LGALS3BP, LRG1, MPO, MRC2, ORM1, PIGR, PON1, TIMP1
3	CADM1, CD163, CDH5, CP, CTSD, F5, FHR3, GOLM1, HLA-A, HYOU1, ICAM1, LGALS3BP, LRG1, MFAP4, MMRN1, MPO, MRC2, ORM1, PON1, PRG4, TIMP1
4	CADM1, CD163, CD44, CP, CTSD, F5, FHR3, FN1, GOLM1, HLA-A, HYOU1, ICAM2, IGHA2, LCN2, LGALS3BP, LRG1, MFAP4, MMRN1, MPO, MRC2, ORM1, PON1, PRG4, TIMP1, VTN
5	CADM1, CD109, CD163, CDH5, CFH, CP, CTSD, F5, FCGBP, FHR3, HLA-A, HYOU1, KDR, LGALS3BP, LRG1, MFAP4, MPO, MRC2, ORM1, PRG4, TIMP1
6	APMAP, CADM1, CD163, CDH5, CFH, CP, CTSD, F5, FHR3, FN1, GOLM1, HLA-A, HP, HYOU1, LCN2, LGALS3BP, LRG1, MPO, MRC2, ORM1, PON1, TIMP1, VTN
7	CADM1, CD163, CDH5, CTSD, F5, FHR3, FN1, HLA-A, HYOU1, LGALS3BP, LRG1, MRC2, PON1, VTN
8	AFM, CADM1, CD163, CTSD, F5, FHR3, FN1, HLA-A, HYOU1, ICAM2, LCN2, LGALS3BP, LRG1, MPO, MRC2, ORM1, PIGR, PON1, PRG4, TIMP1, TNC, VTN

**b**	**Significant proteins selected into logistic regression models by stepwise selection**		
Fold	Predictive logistic regression models	Training (7/8 dataset)	Validation (1/8 dataset)
1	CADM1, CD109, CDH5, FN1, HLA-A, HYOU1, LGALS3BP, LUM, MRC2	0.79	0.58
2	CADM1, CD109, ICAM1, IGJ, LGALS3BP, LRG1, MRC2	0.75	0.60
3	CADM1, CP, HYOU1, LGALS3BP, LRG1, MMRN1, MPO, PRG4	0.77	0.48
4	HYOU1, IGHA2, LGALS3BP, LRG1, MRC2, VTN	0.74	0.58
5	CADM1, CD109, FETUB, HP, HYOU1, LGALS3BP, LRG1	0.77	0.69
6	CADM1, FN1, HLA-A, HYOU1, LGALS3BP, LRG1, MRC2, TIMP1, VTN	0.78	0.63
7	CADM1, FN1, HYOU1, LGALS3BP, VTN	0.72	0.82
8	AFM, CADM1, HYOU1, LGALS3BP, LRG1, VTN	0.77	0.67
Consensus	MRC2, VTN, LRG1, CADM1, HYOU1, LGALS3BP	0.72	

Appendix Table S6. Grading biomarker signature development within 10-fold CV. **a**, Differentially abundant proteins characterised as significant in the individual folds of the training dataset. **b**, Proteins selected into logistic regression models in individual folds. The consensus model contains proteins with a high frequency of occurrence in the individual folds. AUC values are reported.

**a**	**Significant proteins for each fold (FDR<0.05, fold change cut-off ±1.1)**
Fold	Differentially abundant proteins
1	AHSG, ANT3, ATRN, CD109, CDH5, CFH, CLU, DSG2, F11, F5, FETUB, FGA, GOLM1, HPX, HRG,

	IGJ, KLKB1, LCN2, LGALS3BP, LRG1, LUM, LYVE1, MST1, NCAM1, ORM1, PGCP, PLXDC2, PLXNB2, PRG4, SERPINA3, THBS1, VWF
2	A1AG2, AHSG, ANT3, ATRN, BTD, CADM1, CD109, CD44, CFH, CFI, CLU, CTSD, DSG2, FETUB, FGA, GOLM1, HLA-A, HPX, HRG, ICAM2, IGJ, KLKB1, LCN2, LGALS3BP, LUM, LYVE1, MPO, NCAM1, PGCP, PRG4, PROC, SERPINA3, VWF
3	A1AG2, AHSG, ANT3, ATRN, BTD, CADM1, CD109, CD44, CFH, CFI, CLU, CTSD, DKFZp686N02209, DSG2, ECM1, F11, F5, FETUB, FGA, FHR3, FN1, GOLM1, HPX, HRG, IGFBP3, IGHA2, KLKB1, LCN2, LGALS3BP, LRG1, LUM, LYVE1, MPO, MST1, NCAM1, PGCP, PLXNB2, PON1, PRG4, Q5JNX2, SERPINA3, SERPINA6, SERPINA7, VWF
4	A1AG2, AHSG, ANT3, ATRN, BTD, CADM1, CD109, CD44, CFH, CLU, DKFZp686N02209, DSG2, ECM1, FETUB, FGA, GOLM1, HPX, HRG, IGHA2, IGHG2, IGJ, LCN2, LGALS3BP, LUM, LYVE1, MPO, MRC2, MST1, NCAM1, PGCP, PON1, PRG4, SERPINA1, SERPINA3, THBS1, TIMP1, VWF
5	A1AG2, AFM, AHSG, ANT3, ATRN, BTD, CADM1, CD109, CD44, CDH5, CFH, CFI, CLU, CP, CTSD, DSG2, ECM1, F11, F5, FETUB, FGA, FN1, GOLM1, HLA-A, HPX, HRG, IGFBP3, KLKB1, LCN2, LUM, LYVE1, MFAP4, MRC2, MST1, NCAM1, PGCP, PLXNB2, PRG4, Q5JNX2, SERPINA6, SERPINA7, VWF
6	A1AG2, AHSG, ANT3, BTD, CADM1, CD109, CD44, CFH, CLU, DSG2, ECM1, F5, FETUB, FGA, FHR3, GOLM1, HPX, HRG, KLKB1, LCN2, LGALS3BP, LRG1, LUM, LYVE1, MRC2, MST1, NCAM1, PGCP, PLTP, PLXDC2, PLXNB2, PON1, SERPINA3, THBS1, VWF
7	A1AG2, AFM, AHSG, ANT3, BTD, CADM1, CD109, CD44, CFH, CFI, CLU, DKFZp686N02209, DSG2, ECM1, F11, F5, FETUB, FGA, FHR3, FN1, GOLM1, HPX, HRG, IGFBP3, KLKB1, KNG1, LAMP2, LCN2, LRG1, LUM, LYVE1, MST1, NCAM1, PGCP, PLXNB2, PON1, PRG4, Q5JNX2, SERPINA3, SERPINA7, THBS1, VWF
8	AHSG, ANT3, ATRN, BTD, CADM1, CD109, CFH, CFI, CLU, DSG2, F11, FETUB, FGA, FHR3, GOLM1, HPX, HRG, ICAM2, KLKB1, LCN2, LGALS3BP, LRG1, LUM, LYVE1, MRC2, MST1, NCAM1, PGCP, PLXNB2, PON1, PRG4, SERPINA3, VWF
9	AHSG, ANT3, ATRN, BTD, CADM1, CD109, CFH, CLU, DSG2, FETUB, FGA, GOLM1, HLA-A, HP, HPX, HRG, KLKB1, LCN2, LGALS3BP, LUM, LYVE1, MPO, NCAM1, ORM1, PGCP, PLXNB2, PON1, PRG4, SERPINA3, SERPINA7, THBS1, TIMP1, VWF
10	A1AG2, AHSG, ANT3, BTD, CADM1, CD109, CD44, CFH, CFI, CLU, CTSD, DSG2, F11, FETUB, FGA, FHR3, FN1, GOLM1, HPX, HRG, IGFBP3, IGHM, KLKB1, LCN2, LGALS3BP, LUM, LYVE1, MFAP4, MMRN1, MST1, NCAM1, ORM1, PGCP, PLXNB2, PON1, PRG4, SERPINA3, SERPINA7, TNC, VWF

**b**	**Significant proteins selected into logistic regression models by stepwise selection**		
Fold	Predictive logistic regression models	Training (9/10 dataset)	Validation (1/10 dataset)
1	ATRN, CD109, CLU, DSG2, FGA, LGALS3BP, LYVE1, NCAM1, PGCP	0.62	0.42
2	CD109, CD44, CFH, CLU, FGA, HRG, IGJ, KLKB1, LYVE1, MPO, PGCP, PRG4	0.62	0.53
3	CD109, CFH, CLU, CTSD, DSG2, ECM1, LYVE1, MPO, PGCP, PRG4	0.61	0.50
4	CD109, CFH, CLU, DSG2, ECM1, FGA, IGJ, LGALS3BP, LYVE1, NCAM1, PGCP, PRG4, THBS1	0.60	0.70
5	AFM, CD109, CFH, CLU, CTSD, ECM1, HRG, NCAM1, PGCP, PRG4, VWF	0.62	0.61
6	CD109, CLU, ECM1, FGA, KLKB1, LYVE1, NCAM1, PON1, THBS1, VWF	0.55	0.61
7	BTD, CD109, CFH, CLU, DSG2, ECM1, FHR3, KLKB1, KNG1, LYVE1, PGCP, PRG4, THBS1	0.62	0.50
8	CD109, CLU, FGA, KLKB1, LGALS3BP, LYVE1, NCAM1, PGCP, PON1	0.59	0.39
9	ATRN, CD109, CLU, DSG2, FGA, HPX, LYVE1, NCAM1, PGCP, THBS1, VWF	0.62	0.61
10	ANT3, CD109, CFI, CLU, IGFBP3, LYVE1, MFAP4, MST1, NCAM1, ORM1, PGCP, PON1, TNC	0.64	0.39
Consensus	CLU, CD109, LYVE1, PGCP, NCAM1, FGA, PRG4, ECM1		

Appendix Table S7. Clinical stage biomarker signature development within 10-fold CV. **a**, Differentially abundant proteins characterised as significant in the individual folds of the training dataset. **b**, Proteins

selected into logistic regression models in individual folds. The consensus model contains proteins with a high frequency of occurrence in the individual folds. AUC values are reported.

a	Significant proteins for each fold (FDR<0.05, fold change cut-off ±1.1)
Fold	Differentially abundant proteins
1	HLA-A, MPO, CDH5, CFI, LRG1, PIGR, FETUB, F5, FHR3, FN1, CFH, HPX, IGHG2, LGALS3BP, DKFZp686N02209, VWF, MRC2, FCGBP, PLTP, FGG, BTD, IGFBP3, HP, CTSD, AFM, Q5JNX2, MFAP4, NCAM1, DSG2, ICAM2, LYVE1, HRG, CD163, KNG1, KLKB1, CD109, TNC, LUM, AOC3, CLU, FGA, SERPINA3, ATRN, VTN, TIMP1, PON1, CADM1, APMAP, ANT3, F11, LCN2, IGHA2, PLXDC2, ITIH4, IGHM, CD44, HYOU1, ICAM1, THBS1, ECM1, PTPRJ, GOLM1, PRG4, SERPINA6, IGJ, PROC, SERPINA1, PGCP
2	CFI, HPX, PIGR, LRG1, F5, FETUB, HLA-A, AFM, IGHG2, FHR3, DKFZp686N02209, FN1, MRC2, CFH, FCGBP, MPO, THBS1, ICAM2, HRG, BTD, PLTP, AHSG, SERPINA3, VWF, NCAM1, CDH5, LGALS3BP, ATRN, ORM1, FGG, HP, APMAP, AOC3, MFAP4, KNG1, CTSD, Q5JNX2, LYVE1, CLU, CD109, LCN2, LUM, ICAM1, TIMP1, CD163, IGHA2, DSG2, PLXDC2, PTPRJ, VTN, TNC, GOLM1, F11, ANT3, CD44, KLKB1, IGJ, SERPINA1, PGCP, PON1, ITIH4, CADM1, PLXNB2, PROC, FGA, ECM1, PRG4, IGHM, APOB
3	LGALS3BP, MPO, CFI, F5, LRG1, PIGR, HPX, FHR3, FETUB, FN1, FGG, CFH, IGHG2, ICAM2, MRC2, DKFZp686N02209, CDH5, PLTP, AFM, FCGBP, HLA-A, TNC, LYVE1, BTD, KNG1, AHSG, F11, HRG, AOC3, CD163, CLU, THBS1, TIMP1, CD109, VWF, LCN2, NCAM1, KLKB1, PLXDC2, GOLM1, Q5JNX2, CTSD, SERPINA3, MFAP4, HP, APMAP, LUM, ANT3, SERPINA7, IGHA2, DSG2, ATRN, ICAM1, CADM1, PON1, PTPRJ, VTN, ITIH4, PRG4, IGHM, IGJ, PROC, HYOU1, SERPINA6, ORM1, APOB, FGA, CD44, PGCP, IGFBP3, A1AG2, PLXNB2
4	MPO, CFI, PIGR, LRG1, FN1, AFM, F5, FETUB, HPX, CFH, IGHG2, THBS1, HLA-A, DKFZp686N02209, FHR3, FCGBP, ICAM2, PLTP, LGALS3BP, NCAM1, FGG, BTD, CDH5, CLU, MRC2, LUM, PON1, F11, HRG, KNG1, VWF, AHSG, VTN, LYVE1, APMAP, KLKB1, ATRN, LCN2, CD163, AOC3, SERPINA7, HP, ANT3, SERPINA3, TIMP1, APOB, ICAM1, ITIH4, MFAP4, PRG4, Q5JNX2, PLXDC2, PTPRJ, GOLM1, TNC, SERPINA6, DSG2, IGHA2, ORM1, CP, ECM1, CTSD, FGA, PROC, IGHM, HYOU1, IGJ, SERPINA1, IGFBP3, CD44, PLXNB2, CD109, A1AG2
5	LGALS3BP, MPO, CFI, HPX, LRG1, PIGR, DKFZp686N02209, F5, FETUB, IGHG2, ICAM2, FCGBP, MRC2, FN1, CFH, FHR3, PLTP, CDH5, HLA-A, CTSD, AFM, FGG, SERPINA3, ITIH4, LUM, CLU, HRG, THBS1, NCAM1, CD163, VWF, PON1, HP, MFAP4, TNC, TIMP1, SERPINA6, Q5JNX2, CP, SERPINA7, BTD, ICAM1, AOC3, AHSG, CD109, DSG2, IGHA2, LCN2, PTPRJ, ANT3, LYVE1, VTN, ATRN, SERPINA1, ORM1, KLKB1, APMAP, PLXNB2, PLXDC2, IGHM, PROC, IGJ, GOLM1, A1AG2, CADM1, PGCP, FGA, PRG4
6	MPO, CFI, HPX, PIGR, LRG1, FN1, IGHG2, F5, AFM, HLA-A, DKFZp686N02209, ICAM2, FETUB, KNG1, FHR3, LGALS3BP, FGG, PLTP, CFH, CDH5, THBS1, AHSG, HRG, MRC2, FCGBP, NCAM1, LCN2, AOC3, SERPINA3, TNC, HP, BTD, F11, IGHM, VWF, LUM, CLU, ORM1, ANT3, CD163, MFAP4, PON1, PTPRJ, KLKB1, ATRN, APMAP, ICAM1, CD109, FGA, IGHA2, LYVE1, CTSD, MMRN1, PLXDC2, HYOU1, PROC, ITIH4, CP, DSG2, Q5JNX2, GOLM1, SERPINA6, TIMP1, VTN, IGJ, PRG4, CD44, PGCP, CADM1
7	HLA-A, LGALS3BP, MPO, CFI, HPX, PIGR, LRG1, F5, MRC2, DKFZp686N02209, IGHG2, FETUB, FCGBP, FN1, PLTP, CDH5, CFH, AFM, FHR3, MFAP4, CD163, ICAM2, NCAM1, CLU, VWF, FGG, LUM, SERPINA3, HRG, PON1, KLKB1, AOC3, CTSD, DSG2, LYVE1, THBS1, HP, ICAM1, TNC, BTD, PTPRJ, APMAP, Q5JNX2, VTN, F11, IGHA2, LCN2, ITIH4, SERPINA6, PROC, ORM1, CADM1, IGHM, CP, PLXDC2, GOLM1, PLXNB2, FGA, ATRN, SERPINA1, HYOU1, PRG4, IGJ, IGFBP3, A1AG2
8	LGALS3BP, MPO, CFI, F5, HPX, LRG1, PIGR, FETUB, FN1, HLA-A, DKFZp686N02209, IGHG2, ICAM2, CFH, MRC2, FHR3, FCGBP, PLTP, AFM, AHSG, AOC3, CTSD, CD163, VWF, HRG, THBS1, CDH5, CLU, FGG, PON1, TIMP1, IGHA2,<br>HP, MFAP4, LCN2, BTD, ICAM1, KNG1, SERPINA3, Q5JNX2, LUM, KLKB1, SERPINA7, APMAP, TNC, DSG2, PRG4, LYVE1, PTPRJ, ITIH4, SERPINA6, IGHM, ATRN, ANT3, PROC, HYOU1, VTN, CD44, F11, ORM1, PLXDC2, CD109, IGJ, FGA, PLXNB2, CADM1, SERPINA1, GOLM1, PGCP, APOB
9	HLA-A, HPX, LRG1, PIGR, FETUB, CFI, F5, MPO, FN1, DKFZp686N02209, LGALS3BP, IGHG2, AFM, PLTP, FHR3, CFH, MRC2, CDH5, ICAM2, THBS1, FGG, FCGBP, CLU, VWF, PON1, LUM, SERPINA3, HRG, PTPRJ, KNG1, ATRN, NCAM1, ITIH4, TNC, ICAM1, KLKB1, SERPINA7, MFAP4, HP, IGHA2, APMAP, AHSG, PLXDC2, TIMP1, AOC3, F11, VTN, FGA, SERPINA6, DSG2, ANT3, LYVE1, CD109, BTD, CTSD, LCN2, PRG4, PROC, IGHM, SERPINA1, CD163, APOB, GOLM1, HYOU1, IGJ, ORM1
10	MPO, CFI, F5, HPX, LRG1, PIGR, CFH, FETUB, FHR3, LGALS3BP, AFM, FN1, IGHG2, FGG, DKFZp686N02209, FCGBP, HLA-A, MRC2, ICAM2, PLTP, KNG1, ATRN, BTD, ANT3, NCAM1, HRG, THBS1, CD163, AHSG, AOC3, SERPINA6, VWF, CDH5, TIMP1, CLU, PTPRJ, TNC, HP, KLKB1, Q5JNX2, ICAM1, SERPINA7, FGA, LCN2, CTSD, F11, MFAP4, VTN, LYVE1, PLXDC2, LUM, SERPINA3, APMAP, PRG4, ITIH4, CD109, IGHA2, DSG2, APOB, IGJ, PROC, PON1, IGHM, ORM1,

	PLXNB2, ECM1		
**b**	**Significant proteins selected into logistic regression models by stepwise selection**		
Fold	Predictive logistic regression models	Training (9/10 dataset)	Validation (1/10 dataset)
1	CFH, CFI, CLU, FETUB, FGG, FHR3, HP, HRG, ICAM1, ICAM2, ITIH4, KLKB1, LUM, LYVE1, NCAM1, PIGR, PON1, PTPRJ, SERPINA1	0.77	0.73
2	APOB, APMAP, CFI, CLU, ECM1, F5, FETUB, FGG, FHR3, HPX, HRG, ICAM1, ICAM2, LYVE1, NCAM1, PIGR, PTPRJ,Q5JNX2	0.77	0.78
3	AOC3, APOB, BTD, CDH5, CFH, CFI, CLU, FCGBP, FETUB, FGG, FHR3, HRG, ICAM2, IGFBP3, IGJ, KNG1, LGALS3BP, LYVE1, MRC2, PIGR, PON1, PTPRJ, SERPINA3, THBS1	0.82	0.65
4	ANT3, AOC3, APOB, CDH5, CFH, CP, F11, FCGBP, FETUB, FGG, FHR3, HP, HRG, ICAM2, IGFBP3, KLKB1, KNG1, LYVE1, MRC2, PIGR, PON1, PROC, PTPRJ, SERPINA1, TIMP1	0.75	0.76
5	AHSG, ANT3, ATRN, CDH5, CFH, CP, FCGBP, FETUB, FGG, FHR3, FN1, HRG, ICAM1, ICAM2, IGHM, IGJ, KLKB1, LUM, LYVE1, MRC2, NCAM1, PIGR, PLXNB2, PON1, PTPRJ, SERPINA1, SERPINA3, SERPINA6, THBS1	0.78	0.64
6	CFH, CLU, CP, FCGBP, FETUB, FGG, FHR3, GOLM1, HP, HRG, ICAM2, KLKB1, KNG1, LYVE1, MRC2, PIGR, PON, PTPRJ	0.72	0.69
7	CFI, CLU, CP, FETUB, FGG, HPX, ICAM1, ICAM2, IGFBP3, KLKB1, LYVE1, PIGR, PROC, PTPRJ, SERPINA1, SERPINA3	0.73	0.76
8	APOB, APMAP, CDH5, CFH, CFI, CLU, FETUB, FGG, FHR3, HLA-A, HP, HRG, ICAM1, ICAM2, KNG1, LYVE1, MPO, PIGR, PON1, PTPRJ, SERPINA1, SERPINA6, THBS1	0.75	0.71
9	AOC3, APOB, CFH, CFI, CLU, FETUB, FHR3, HP, HPX, ICAM1, IGHG2, IGJ, LGALS3BP, LYVE1, ORM1, PIGR, PON1, PTPRJ, SERPINA1, SERPINA3, SERPINA6, VWF	0.77	0.57
10	AOC3, APOB, BTD, CD163, CFH, CLU, ECM1, FGCBP, FETUB, FGG, FHR3, HRG, ICAM2, IGJ, ITIH4, KNG1, LGALS3BP, LYVE1, MRC2, PIGR, PON1, PTPRJ, TIMP1	0.77	0.67
Consensus	FETUB, PIGR, LYVE1, PTPRJ, FGG, FHR3, ICAM2, PON1, HRG, CLU, CFH, SERPINA1, ICAM1, CFI, APOB		

Appendix Table S8. Disseminated disease biomarker signature development within 10-fold CV. **a**, Differentially abundant proteins characterised as significant in the individual folds of the training dataset. **b**, Proteins selected into logistic regression models in individual folds. The consensus model contains proteins with a high frequency of occurrence in the individual folds. AUC values are reported. The median AUC presented in figure 2 is in bold. **c**, Parameters of the consensus model, the linear combination of proteins, and the selected cutoff for disseminated disease prediction.

**a**	**Significant proteins for each fold (FDR<0.05, fold change cut-off ±1.1)**
Fold	Differentially abundant proteins
1	AFM, ATRN, APMAP, CD109, CDH5, CFH, CFI, CP, CTSD, DKFZp686N02209, F5, FCGBP, FETUB, FGA, FGG, FHR3, FN1, HPX, HYOU1, IGFBP3, IGHG2, IGJ, KLKB1, KNG1, LRG1, MPO, MRC2, NCAM1, ORM1, PIGR, PLTP, PLXDC2, PRG4, PROC, PTPRJ, SERPINA3, TNC, VTN, VWF
2	APMAP, CADM1, CD109, CFH, CFI, DKFZp686N02209, F5, FCGBP, FETUB, FGG, FHR3, FN1, HPX, HYOU1, ICAM2, IGHG2, ITIH4, KDR, LRG1, LUM, MPO, MRC2, NCAM1, PGCP, PIGR, PLTP, PLXDC2, PON1, PTPRJ, Q5JNX2, SERPINA3, TIMP1, TNC, VTN
3	AFM, APMAP, CD109, CDH5, CFH, CFI, CLU, CTSD, DKFZp686N02209, F5, FCGBP, FETUB, FGA,

	FGG, FHR3, FN1, HPX, HYOU1, ICAM1, IGHG2, IGJ, ITIH4, KDR, KLKB1, LRG1, LUM, MPO, MRC2, NCAM1, PIGR, PLTP, PLXDC2, PON1, PROC, PTPRJ, SERPINA1, SERPINA3, TIMP1, TNC
4	AFM, APMAP, CFH, CFI, CLU, DKFZp686N02209, F5, FCGBP, FETUB, FGA, FGG, FHR3, FN1, HPX, HYOU1, IGHG2, ITIH4, KDR, KLKB1, LRG1, LUM, MPO, MRC2, NCAM1, PIGR, PLTP, PLXDC2, PON1, PROC, PTPRJ, SERPINA3, TNC, VTN
5	AFM, APMAP, CFH, CFI, DKFZp686N02209, F5, FCGBP, FETUB, FGA, FGG, FHR3, FN1, HP, HPX, HYOU1, ICAM1, IGHG2, IGJ, ITIH4, KDR, KLKB1, LRG1, LUM, MPO, NCAM1, PIGR, PLTP, PLXDC2, PON1, PROC, PTPRJ, SERPINA1, SERPINA3, THBS1, TIMP1, TNC, VTN
6	AFM, APMAP, CD109, CFH, CFI, CLU, CTSD, DKFZp686N02209, F5, FCGBP, FETUB, FGA, FGG, FHR3, FN1, HP, HPX, HYOU1, ICAM1, IGHG2, IGHM, IGJ, ITIH4, KDR, KLKB1, LRG1, LUM, MMRN1, MPO, MRC2, NCAM1, PIGR, PLTP, PLXDC2, PON1, PROC, PTPRJ, SERPINA3, THBS1, TNC, VTN
7	APMAP, CFH, CFI, CLU, DKFZp686N02209, F5, FCGBP, FETUB, FGA, FGG, FHR3, FN1, HPX, HYOU1, IGHG2, ITIH4, KDR, KLKB1, LRG1, LUM, MRC2, NCAM1, PIGR, PLTP, PLXDC2, PON1, PTPRJ, SERPINA3, THBS1, TNC, VTN
8	AFM, APMAP, CD109, CFH, CFI, DKFZp686N02209, F5, FCGBP, FETUB, FGA, FGG, FHR3, FN1, HPX, HYOU1, IGHA2, IGHG2, ITIH4, KDR, KLKB1, LRG1, LUM, MPO, MRC2, PIGR, PLTP, PLXDC2, PON1, PROC, PTPRJ, SERPINA3, THBS1, TIMP1, TNC, VTN
9	AFM, APMAP, CD109, CFH, CFI, DKFZp686N02209, F5, FCGBP, FETUB, FGA, FGG, FHR3, FN1, HLA-A, HPX, HYOU1, IGHG2, ITIH4, KDR, KLKB1, LRG1, LUM, MPO, MRC2, NCAM1, PIGR, PLTP, PLXDC2, PON1, PROC, PTPRJ, SERPINA3, THBS1, TIMP1, TNC, VTN
10	AFM, APMAP, CD109, CFH, CFI, CLU, DKFZp686N02209, F5, FCGBP, FETUB, FGA, FGG, FHR3, FN1, GOLM1, HP, HPX, HYOU1, IGHG2, ITIH4, KDR, KLKB1, LCN2, LRG1, LUM, MPO, MRC2, NCAM1, PIGR, PLTP, PLXDC2, PON1, PROC, PTPRJ, Q5JNX2, SERPINA3, THBS1, TIMP1, TNC, VTN

**b**	**Significant proteins selected into logistic regression models by stepwise selection**		
Fold	Predictive logistic regression models	Training (9/10 dataset)	Validation (1/10 dataset)
1	CFH, F5, FETUB, IGHG2, IGJ, KNG1, MPO, PIGR, PTPRJ, VTN	0.92	0.67
2	CFH, DKFZp686N02209, FGG, IGHG2, PIGR, PTPRJ, VTN	0.90	**0.82**
3	CDH5, CFH, CFI, CLU, DKFZp686N02209, F5, FGG, IGJ, ITIH4, LRG1, MPO, PIGR, PTPRJ, SERPINA1	0.92	0.85
4	CFH, CLU, F5, FGG, ITIH4, NCAM1, PIGR, PLXDC2, PON1, PTPRJ, VTN	0.91	0.92
5	AFM, CFH, DKFZp686N02209, F5, FGG, ICAM1, ITIH4, KLKB1, PIGR, PON1, PTPRJ, SERPINA1, THBS1	0.92	0.97
6	CFH, CLU, F5, FETUB, FGG, IGHG2, IGHM, IGJ, ITIH4, MMRN1, MPO, NCAM1, PIGR, PTPRJ, VTN	0.94	0.80
7	F5, FETUB, FGA, FGG, IGHG2, NCAM1, PIGR, PTPRJ, VTN	0.92	0.71
8	AFM, CFH, DKFZp686N02209, F5, FETUB, FGG, MPO, PIGR, PLXDC2, PTPRJ, THBS1, VTN	0.91	0.92
9	CFH, CFI, F5, FETUB, FGA, FGG, FHR3, HLA-A, IGHG2, ITIH4, KDR, KLKB1, LUM, NCAM1, PIGR, PLTP, PTPRJ, SERPINA3, THBS1	0.93	0.63
10	AFM, CFH, F5, FGG, IGHG2, LCN2, PIGR, PLTP, PTPRJ, VTN	0.91	0.75
Consensus	PTPRJ, PIGR, CFH, F5, FGG, VTN, IGHG2, ITIH4, FETUB		0.90

**c**	**Consensus model** where stage 4=1, stages 1+2+3=0
Model	-27.9437633 - 1.1032118*PTPRJ + 1.0899420*PIGR + 0.7643993*CFH + 0.3666503*F5 - 1.0169896*FGG - 1.0493068*VTN + 0.7813780*IGHG2 + 0.5811620*ITIH4 + 0.6886607*FETUB
Cutoff	0.256

Appendix Table S9. Reproducibility assessment of the disseminated disease biomarker signature within 8-fold CV. **a**, Differentially abundant proteins characterised as significant in the individual folds of the training dataset. **b**, Proteins selected into logistic regression models in individual folds. The consensus model contains proteins with a high frequency of occurrence in the individual folds. AUC values are reported.

**a**	**Significant proteins for each fold (FDR<0.05, fold change cut-off ±1.1)**
Fold	Differentially abundant proteins
1	APMAP, CD109, CFH, CFI, CLU, DKFZp686N02209, F5, FCGBP, FETUB, FGA, FGG, FHR3, FN1, HPX, ICAM1, IGFBP3, IGHG2, IGJ, LRG1, LUM, MPO, MRC2, NCAM1, PIGR, PLTP, PLXDC2, PON1, PROC, PTPRJ, Q5JNX2, SERPINA3, THBS1, TNC, VTN, VWF
2	AFM, ATRN, APMAP, CD109, CFH, CFI, DKFZp686N02209, F5, FCGBP, FETUB, FGA, FGG, FHR3, FN1, GOLM1, HP, HPX, HYOU1, IGFBP3, IGHG2, ITIH4, KDR, LRG1, LUM, MPO, MRC2, NCAM1, PIGR, PLTP, PLXDC2, PON1, PROC, PTPRJ, SERPINA3, THBS1, TIMP1, TNC, VTN
3	AFM, AOC3, ATRN, APMAP, CD109, CFH, CFI, DKFZp686N02209, F5, FCGBP, FETUB, FGA, FGG, FHR3, FN1, HPX, HYOU1, IGFBP3, IGHG2, IGHM, IGJ, KDR, LRG1, MRC2, NCAM1, PIGR, PLTP, PLXDC2, PRG4, PROC, PTPRJ, SERPINA3, TNC, VTN
4	AFM, APMAP, CD163, CDH5, CFH, CFI, DKFZp686N02209, F5, FCGBP, FETUB, FGA, FGG, FHR3, FN1, HP, HPX, HYOU1, IGFBP3, IGHA2, IGHG2, ITIH4, KDR, LRG1, LUM, MMRN1, MPO, MRC2, NCAM1, PGCP, PIGR, PLTP, PLXDC2, PON1, PROC, PTPRJ, SERPINA3, THBS1, TIMP1, TNC, VTN
5	AFM, AOC3, APMAP, CD109, CFH, CFI, CLU, DKFZp686N02209, F5, FCGBP, FETUB, FGA, FGG, FHR3, FN1, HP, HPX, HYOU1, ICAM1, IGFBP3, IGHG2, ITIH4, KDR, LGALS3BP, LRG1, LUM, MPO, NCAM1, PIGR, PLTP, PLXDC2, PON1, PROC, PTPRJ, SERPINA1, SERPINA3, THBS1, TIMP1, TNC, VTN
6	A1AG2, APMAP, CADM1, CD109, CFH, CFI, CLU, CTSD, DKFZp686N02209, F5, FCGBP, FETUB, FGA, FGG, FHR3, FN1, HPX, HYOU1, ICAM1, IGFBP3, IGHG2, IGJ, ITIH4, KDR, LGALS3BP, LRG1, LUM, LYVE1, MPO, MRC2, PIGR, PLTP, PLXDC2, PON1, PROC, PTPRJ, SERPINA3, SERPINA7, THBS1, TIMP1, TNC, VTN, VWF
7	AFM, APMAP, CFH, CFI, CLU, DKFZp686N02209, F5, FCGBP, FETUB, FGA, FGG, FHR3, FN1, HPX, HYOU1, IGFBP3, IGHG2, ITIH4, KDR, LRG1, LUM, MPO, MRC2, NCAM1, PIGR, PLTP, PLXDC2, PON1, PROC, PTPRJ, SERPINA3, TIMP1, TNC, VTN
8	AFM, APMAP, CD109, CFH, CFI, DKFZp686N02209, F5, FCGBP, FETUB, FGA, FGG, FHR3, FN1, HPX, HYOU1, IGFBP3, IGHG2, ITIH4, KDR, LRG1, LUM, MPO, MRC2, PIGR, PLTP, PLXDC2, PON1, PROC, PTPRJ, Q6N091, SERPINA3, TNC, VTN

**b**	**Significant proteins selected into logistic regression models by stepwise selection**		
Fold	Predictive logistic regression models	Training (7/8 dataset)	Validation (1/8 dataset)
1	CFH, F5, FETUB, FGG, IGHG2, IGJ, MPO, NCAM1, PIGR, PTPRJ, VTN	0.92	0.84
2	AFM, CFH, FETUB, FGG, IGHG2, ITIH4, PIGR, PLXDC2, PTPRJ, VTN	0.90	0.91
3	AFM, CFH, F5, FETUB, FGG, IGHG2, IGJ, PIGR, PTPRJ, VTN	0.92	0.81
4	AFM, CDH5, CFH, F5, FGA, FGG, IGHG2, MMRN1, NCAM1, PIGR, PTPRJ, VTN	0.91	0.67
5	CLU, FETUB, HPX, ITIH4, NCAM1, PIGR, PLTP, PTPRJ, SERPINA1, VTN	0.92	0.58
6	APMAP, CFH, DKFZp686N02209, F5, FGG, IGHG2, IGJ, LYVE1, PIGR, PTPRJ, THBS1, VTN	0.95	0.61
7	CFH, CFI, F5, FETUB, FGG, IGHG2, NCAM1, PIGR, PTPRJ, VTN	0.92	0.78
8	AFM, CFH, FGG, IGHG2, PIGR, PON1, PTPRJ, VTN	0.89	0.93
Consensus	AFM, NCAM1, F5, FETUB, CFH, FGG, IGHG2, PIGR, PTPRJ, VTN		0.91

Appendix Table S10. Forced selection of clinical factors (i.e. age, gender, and stage) into predictive biomarker signatures for **a**, regional localization, and **b**, TNM metastasis status. Proteins selected into logistic regression models in individual folds and the corresponding AUC values are reported. The consensus model

contains proteins with a high frequency of occurrence in the individual folds.

a	Significant proteins selected into regional localization models by stepwise selection		
Fold	Predictive logistic regression models	Training (9/10 dataset)	Validation (1/10 dataset)
1	Age, Gender, Stage, CADM1, FN1, HRG, HYOU1, LGALS3BP, LRG1, MRC2, TIMP1, VTN	0.81	0.52
2	Age, Gender, Stage, CADM1, FN1, HYOU1, LGALS3BP, VTN	0.78	0.67
3	Age, Gender, Stage, CADM1, FN1, HYOU1, LGALS3BP, VTN	0.76	0.77
4	Age, Gender, Stage, CADM1, CD109, LGALS3BP, LRG1	0.79	0.67
5	Age, Gender, Stage, CADM1, CD109, HYOU1, LGALS3BP, LRG1, TIMP1	0.82	0.56
6	Age, Gender, Stage, CADM1, FN1, HYOU1, LGALS3BP, MRC2, VTN	0.79	0.49
7	Age, Gender, Stage, CADM1, CD109, HYOU1, LGALS3BP, LRG1, TIMP1	0.79	0.79
8	Age, Gender, Stage, CADM1, FN1, HYOU1, LGALS3BP, VTN	0.77	0.74
9	Age, Gender, Stage, CADM1, FN1, HP, HYOU1, LGALS3BP, LRG1, MMRN1, VTN	0.79	0.81
10	Age, Gender, Stage, CADM1, FN1, HYOU1, LGALS3BP	0.76	0.64
Consensus	Age, Gender, Stage, LGALS3BP, CADM1, HYOU1, FN1, VTN, LRG1		0.77
**b**	**Significant proteins selected into TNM metastasis status models by stepwise selection**		
Fold	Predictive logistic regression models	Training (9/10 dataset)	Validation (1/10 dataset)
1	Age, Gender, APMAP, CFH, F5, FETUB, FGG, IGHG2, IGJ, KNG1, MPO, NCAM1, PIGR, PLTP, PLXDC2, PRG4, PTPRJ, VTN	0.94	0.64
2	Age, Gender, CFH, CFI, DKFZp686N02209, F5, FGG, IGHG2, MRC2, NCAM1, PIGR, PTPRJ, Q5JNX2, VTN	0.92	0.77
3	Age, Gender, CDH5, CFH, CFI, CLU, DKFZp686N02209, F5, FGA, FGG, IGHG2, IGJ, ITIH4, KLKB1, LRG1, MPO, PIGR, PTPRJ, SERPINA1	0.93	0.83
4	Age, Gender, AFM, CFH, F5, FGG, IGHG2, NCAM1, PIGR, PON1, PTPRJ, VTN	0.90	0.95
5	Age, Gender, AFM, CFH, DKFZp686N02209, F5, FGA, FGG, ICAM1, IGHG2, KLKB1, MPO, PIGR, PLTP, PTPRJ, SERPINA1	0.92	0.92
6	Age, Gender, AFM, CFH, CLU, F5, FETUB, FGG, IGHG2, IGJ, ITIH4, MMRN1, MPO, PIGR, PTPRJ, SERPINA3, VTN	0.94	0.79
7	Age, Gender, F5, FETUB, FGA, FGG, IGHG2, NCAM1, PIGR, PTPRJ, VTN	0.92	0.69
8	Age, Gender, AFM, CFH, F5, FETUB, FGG, IGHG2, MPO, PIGR, PLXDC2, PON1, PTPRJ, VTN	0.91	0.88
9	Age, Gender, AFM, CFH, DKFZp686N02209, F5, FGA, FGG, HLA-A, IGHG2, KLKB1, LUM, PIGR, PLTP, PTPRJ, SERPINA3	0.93	0.69
10	Age, Gender, AFM, CFH, F5, FGG, IGHG2, LCN2, PIGR, PLTP, PTPRJ, VTN	0.91	0.77
Consensus	Age, Gender, PTPRJ, PIGR, IGHG2, FGG, F5, CFH, VTN, AFM, MPO		0.90

Appendix Table S11. The performance of individual outcome signature proteins on the protein or transcript levels. The predictive ability of each predictor at a time was assessed within cross validation (CV) **a**, in the SRM proteomic data set associated with overall survival (OS), **b**, in the GSE17536 transcriptomic data set associated with OS, and **c**, in the GSE14333 transcriptomic data set associated with disease-free survival (DFS). AUCfull denotes an upper level of performance reported for the predictive model on the full data set. AUCmedian represents an unbiased performance derived from the pseudomedian fold of the cross-validation.

	**a**	**b**	**c**
Signature proteins	**SRM data set**<br>**OS, 10-fold CV**<br>AUCfull, AUCmedian	**GSE17536 data set**<br>**OS, 5-fold CV**<br>AUCfull, AUCmedian	**GSE14333 data set**<br>**DFS, 10-fold CV**<br>AUCfull, AUCmedian
HLA-A	0.55, 0.57	0.59, 0.6	0.51, 0.5
CFH	0.56, 0.61	0.62, 0.67	0.68, 0.77
CD44	0.64, 0.67	0.56, 0.60	0.52, 0.5
PTPRJ	0.62, 0.71	0.59, 0.55	0.53, 0.58
HP	0.59, 0.58	0.58, 0.61	0.51, 0.58
CDH5	0.52, 0.56	0.59, 0.59	0.58, 0.63

Appendix Table S12. Classification of colon cancer subtypes (CCSs) based on the outcome signature proteins within 10-fold cross-validation of the GSE33113 data set.

			subgroups from paper		
			CCS1	CCS2	CCS3
fold1	predicted by our proteins	CCS1	4	1	0
		CCS2	0	1	0
		CCS3	1	1	3
fold2	predicted by our proteins	CCS1	3	2	0
		CCS2	2	1	0
		CCS3	0	0	3
fold3	predicted by our proteins	CCS1	3	2	0
		CCS2	2	0	1
		CCS3	0	0	2
fold4	predicted by our proteins	CCS1	4	1	0
		CCS2	0	0	0
		CCS3	1	1	3
fold5	predicted by our proteins	CCS1	3	1	0
		CCS2	1	1	0
		CCS3	0	0	2
fold6	predicted by our proteins	CCS1	3	1	1
		CCS2	1	1	0
		CCS3	0	0	1
fold7	predicted by our proteins	CCS1	3	1	0
		CCS2	1	0	0
		CCS3	0	1	2
fold8	predicted by our proteins	CCS1	3	1	1
		CCS2	1	1	0
		CCS3	0	0	1
fold9	predicted by our proteins	CCS1	3	2	1
		CCS2	1	0	0
		CCS3	0	0	1
fold10	predicted by our proteins	CCS1	4	1	1
		CCS2	0	1	0
		CCS3	0	0	1

Appendix Table S13. Classification of the five cellular phenotype subtypes based on the outcome signature proteins within **a**, 5-fold cross-validation of the GSE13294 data set, and **b**, 10-fold cross-validation of the GSE14333 data set.

a			subgroups from paper				
			enterocyte	goblet-like	inflammatory	stem-like	TA
fold1	predicted by our proteins	enterocyte	0	0	0	1	0
		goblet-like	0	3	1	2	1
		inflammatory	2	2	2	0	3
		stem-like	1	0	3	1	0
		TA	2	0	1	0	4
fold2	predicted by our proteins	enterocyte	1	1	1	1	0
		goblet-like	0	0	0	0	1
		inflammatory	3	2	3	2	1
		stem-like	0	0	0	1	0
		TA	1	1	3	0	5
fold3	predicted by our proteins	enterocyte	1	1	0	2	2
		goblet-like	1	0	0	0	1
		inflammatory	1	1	3	1	0
		stem-like	2	0	2	1	0
		TA	0	2	2	0	4
fold4	predicted by our proteins	enterocyte	2	0	3	2	1
		Goblet-like	0	1	0	0	1
		Inflammatory	0	0	1	1	1
		Stem-like	0	1	2	1	0
		TA	3	2	1	0	4
fold5	predicted by our proteins	enterocyte	2	0	1	0	0
		Goblet-like	0	1	0	0	2
		Inflammatory	1	1	5	4	1
		Stem-like	1	0	0	0	0
		TA	0	2	0	0	4

b			subgroups from paper				
			enterocyte	goblet-like	inflammatory	stem-like	TA
fold1	predicted by our proteins	enterocyte	0	0	1	0	0
		goblet-like	0	1	0	0	1
		inflammatory	1	1	1	0	2
		stem-like	1	0	0	3	1
		TA	1	1	1	1	1
fold2	predicted by our proteins	enterocyte	3	0	0	0	1
		goblet-like	0	2	0	0	0
		inflammatory	0	1	1	0	0
		stem-like	0	0	1	2	1
		TA	0	0	1	2	3
fold3	predicted by our proteins	enterocyte	1	0	0	0	0
		goblet-like	0	1	1	0	0

		inflammatory	0	0	0	0	2
		stem-like	1	0	2	4	0
		TA	1	2	0	0	3
fold4	predicted by our proteins	enterocyte	1	0	0	0	0
		goblet-like	0	0	1	0	1
		inflammatory	0	0	1	2	0
		stem-like	0	0	0	2	1
		TA	2	3	1	0	2
fold5	predicted by our proteins	enterocyte	2	0	0	1	0
		goblet-like	0	1	0	0	1
		inflammatory	0	0	1	0	1
		stem-like	0	0	2	2	0
		TA	0	1	0	0	2
fold6	predicted by our proteins	enterocyte	0	1	1	0	0
		goblet-like	0	1	0	0	0
		inflammatory	0	0	1	0	0
		stem-like	1	0	1	3	0
		TA	1	0	0	0	4
fold7	predicted by our proteins	enterocyte	1	0	2	0	2
		goblet-like	0	1	0	0	0
		inflammatory	0	0	1	0	1
		stem-like	1	1	0	3	0
		TA	0	0	0	0	1
fold8	predicted by our proteins	enterocyte	1	0	0	0	1
		goblet-like	1	0	0	1	2
		inflammatory	0	0	1	0	0
		stem-like	0	0	0	2	0
		TA	0	2	1	0	1
fold9	predicted by our proteins	enterocyte	1	0	0	0	1
		goblet-like	0	0	2	0	0
		inflammatory	1	1	0	2	1
		stem-like	0	0	0	1	0
		TA	0	1	0	0	2
fold10	predicted by our proteins	enterocyte	2	0	1	0	1
		goblet-like	0	2	0	0	0
		inflammatory	0	0	1	1	0
		stem-like	0	0	0	2	0
		TA	0	0	0	0	3

Appendix Table S14. The performance of individual localization signature proteins on the protein or transcript levels. The predictive ability of each predictor at a time was assessed within cross validation (CV) **a**, in the SRM proteomic data set, and **b**, in the TCGA transcriptomic data set. AUCfull denotes an upper level of performance reported for the predictive model on the full data set. AUCmedian represents an unbiased performance derived from the pseudomedian fold of the cross-validation.

	**a**	**b**
Signature proteins	**SRM data set**<br>**10-fold CV**<br>AUCfull, AUCmedian	**TCGA**<br>**10-fold CV**<br>AUCfull, AUCmedian
CADM1	0.62, 0.63	0.51, 0.58
LGALS3BP	0.66, 0.71	0.55, 0.54
HYOU1	0.61, 0.68	0.53, 0.56

FN1	0.55, 0.64	0.57, 0.55
VTN	0.57, 0.59	0.52, 0.59
LRG1	0.59, 0.59	0.52, 0.60
MRC2	0.54, 0.58	0.51, 0.57

Appendix Table S15. Functional annotation of signature proteins with gene ontology (GO) biological process terms. **a**, Detailed protein annotation with individual GO terms. **b**, Protein annotation with summarized GO terms that were collapsed into four main categories.

a

Accession	Gene name	GO Biological Process
Q9BY67	CADM1	signal transduction;cell adhesion
P16070	CD44	immune system process;cell adhesion
P33151	CDH5	cell adhesion
P08603	CFH	complement activation;signal transduction;cell adhesion;proteolysis;innate immune response
P00450	CP	copper ion transport;transmembrane transport
P12259	F5	immune system process;cell adhesion;proteolysis;signal transduction;angiogenesis
Q9UGM5	FETUB	endopeptidase activity
P02679	FGG	signal transduction;cell adhesion
P02751	FN1	signal transduction;cell adhesion;acute-phase response;angiogenesis
P01892	HLA-A	B cell mediated immunity;cellular defense response; regulation of immune response
P00738	HP	complement activation;proteolysis;response to stress;acute-phase response
Q9Y4L1	HYOU1	immune system process;cellular protein metabolic process
P01859	IGHG2	complement activation;innate immune response
Q14624	ITIH4	proteolysis;acute-phase response;endopeptidase activity;hyaluronan metabolic process
Q08380	LGALS3BP	macrophage activation;extracellular transport;apoptosis;signal transduction;cell adhesion;proteolysis;cellular defense response
P02750	LRG1	immune system process;cytokine-mediated signaling;cell adhesion
Q9UBG0	MRC2	macrophage activation;intracellular protein transport
P01833	PIGR	B cell mediated immunity;intracellular protein transport
P27169	PON1	peroxidase activity;immune system process;phosphate metabolic process
Q12913	PTPRJ	MAPK activity;T cell receptor signaling;cell migration;cell proliferation;EGFR signaling;protein tyrosine phosphatase activity
P01011	SERPINA3	proteolysis;acute-phase response;inflammatory response; maintenance of gastrointestinal epithelium;endopeptidase activity
P01033	TIMP1	proteolysis;endopeptidase activity;apoptosis;cell migration;cell proliferation
P04004	VTN	cell adhesion;signal transduction;innate immune response;endopeptidase activity;cell migration;vascular endothelial growth factor receptor signaling;complement activation

b

Accession	Gene name	GO Biological Process
Q9BY67	CADM1	cell adhesion(1);signal transduction(2)
P16070	CD44	cell adhesion(1);immune system process(3)
P33151	CDH5	cell adhesion(1)
P08603	CFH	cell adhesion(1);signal transduction(2);immune system process(3);complement activation(3);proteolysis(4)
P00450	CP	transport(2)
P12259	F5	cell adhesion(1);angiogenesis(1);signal transduction(2);immune system process(3);proteolysis(4)
Q9UGM5	FETUB	endopeptidase activity(4)
P02679	FGG	cell adhesion(1);signal transduction(2)
P02751	FN1	cell adhesion(1);angiogenesis(1);signal transduction(2);inflammatory response(3)
P01892	HLA-A	immune system process(3)
P00738	HP	inflammatory response(3);complement activation(3);proteolysis(4)
Q9Y4L1	HYOU1	metabolic process(2);immune system process(3)
P01859	IGHG2	immune system process(3);complement activation(3)
Q14624	ITIH4	metabolic process(2);inflamatory response(3);proteolysis(4);endopeptidase activity(4)
Q08380	LGALS3BP	cell adhesion(1);apoptosis(1);signal transduction(2);transport(2);immune system

		process(3);proteolysis(4)
P02750	LRG1	cell adhesion(1);signaling transduction(2);immune system process(3)
Q9UBG0	MRC2	transport(2);immune system process(3)
P01833	PIGR	transport(2);immune system process(3)
P27169	PON1	metabolic process(2);immune system process(3);peroxidase activity(4)
Q12913	PTPRJ	migration(1);proliferation(1);signal transduction(2)
P01011	SERPINA3	maintenance of gastrointestinal epithelium(1);inflammatory response(3);proteolysis(4);endopeptidase activity(4)
P01033	TIMP1	migration(1);proliferation(1);apoptosis(1);proteolysis(4);endopeptidase activity(4)
P04004	VTN	cell adhesion(1);migration(1);signal transduction(2);immune system process(3);complement activation(3);endopeptidase activity(4)

GO groups: (1) cell adhesion/migration/angiogenesis/proliferation/apoptosis/(maintenance of gastrointestinal epithelium); (2) signal transduction/transport/(metabolic process); (3) immune system process/inflammatory response/(complement activation); (4) proteolysis/endopeptidase activity/(peroxidase activity)

Appendix Figure S1

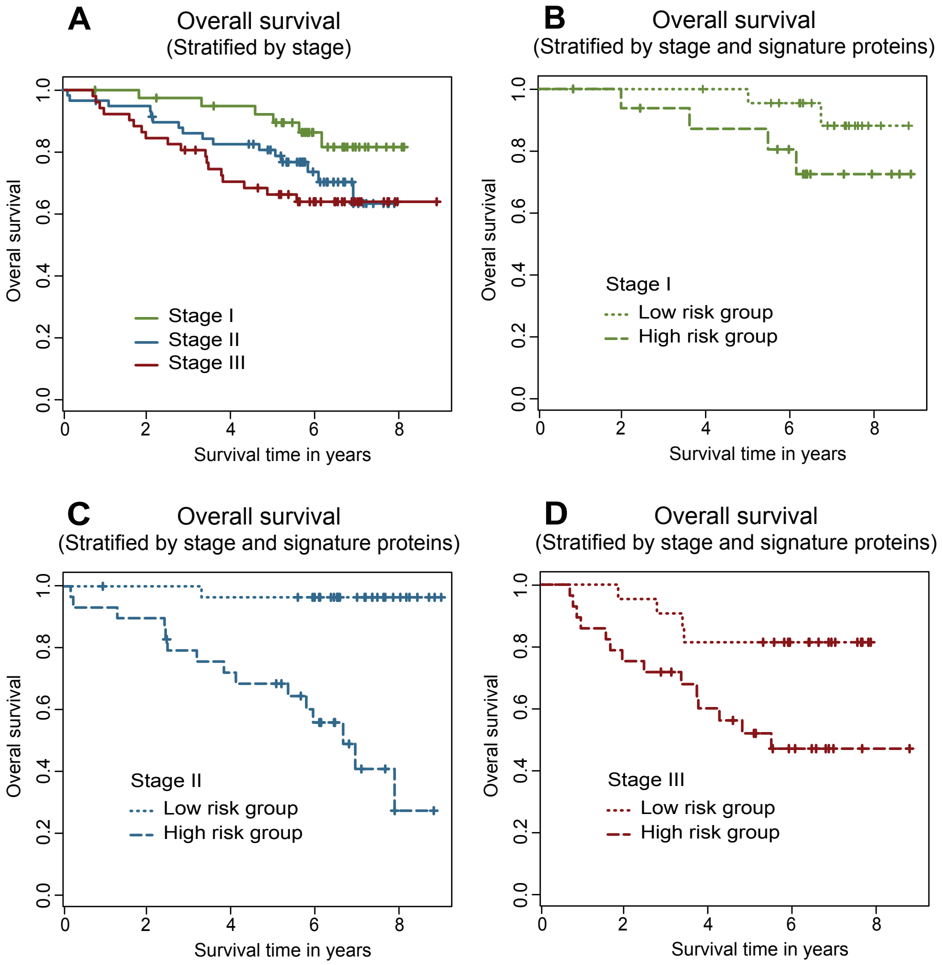

Appendix Figure S2

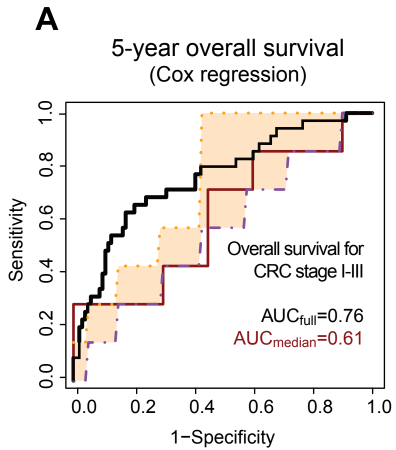

GSE17536 transcriptomic data set

Clinical factors (age, gender, stage), HLA-A, CFH, CD44, PTPRJ, HP, CDH5

Fold	AUC$_{TRAIN}$	AUC$_{LEFT-OUT}$
1	0.72	0.83
2	0.75	0.72
3	0.77	0.59
4	0.78	0.61
5	0.80	0.50

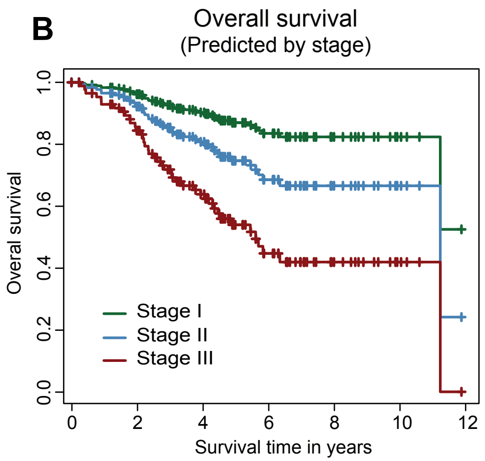

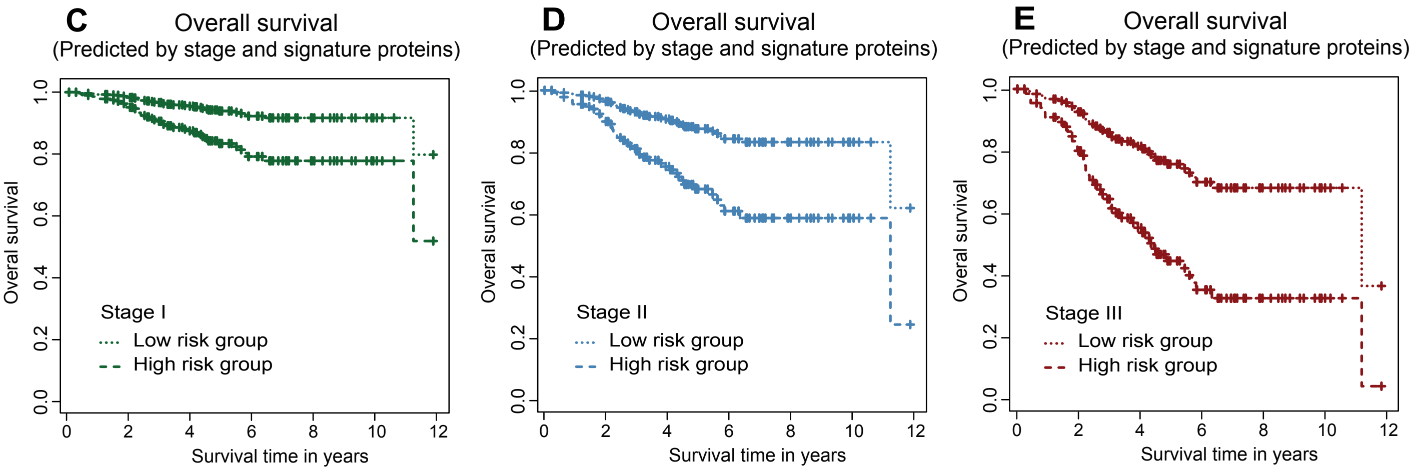

Appendix Figure S3

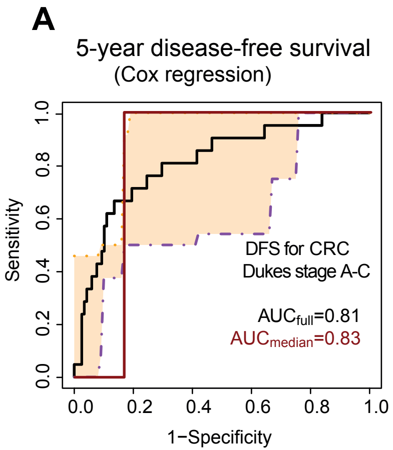

GSE14333 transcriptomic data set

Clinical factors (age, gender, stage), HLA-A, CFH, CD44, PTPRJ, HP, CDH5

Fold	AUC TRAIN	AUC LEFT-OUT
1	0.81	0.67
2	0.81	0.88
3	0.81	0.79
4	0.88	0.33
5	0.81	0.58
6	0.83	0.63
7	0.81	0.83
8	0.80	0.92
9	0.81	0.91
10	0.80	0.91

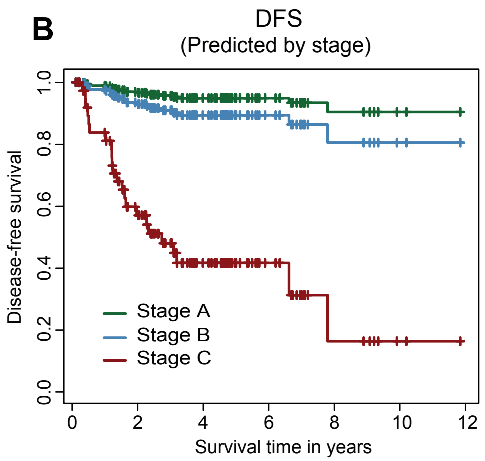

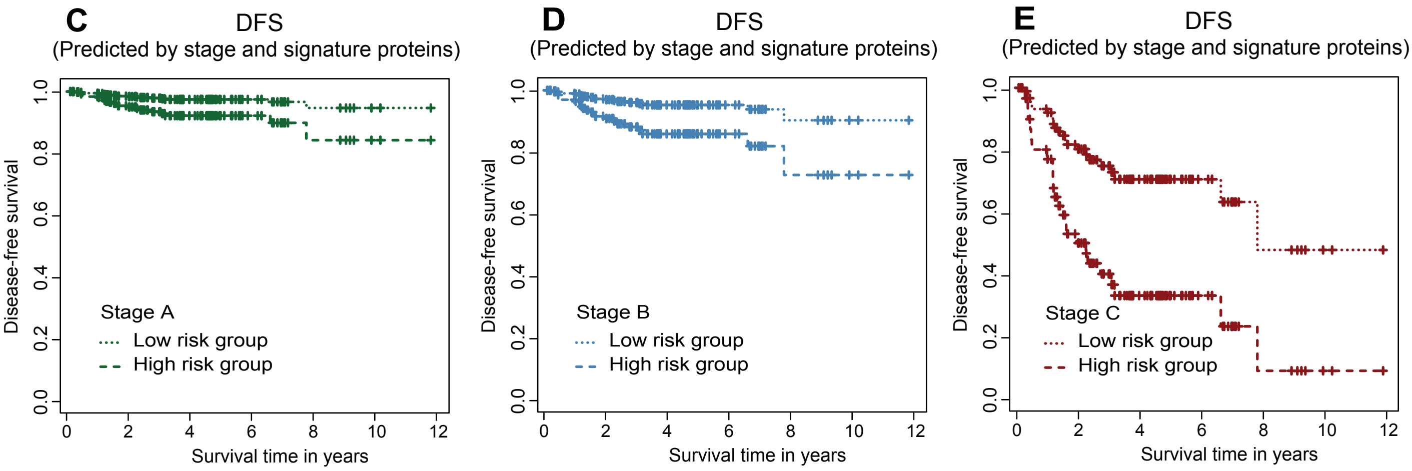

Appendix Figure S4

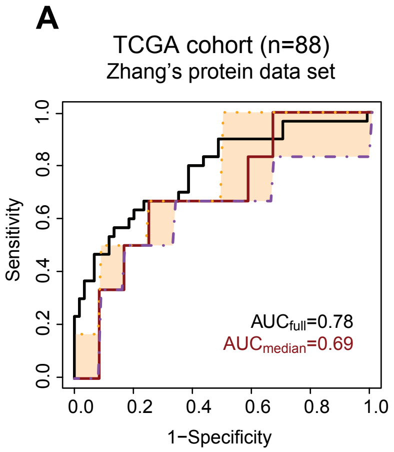

Fold	AUCTRAIN	AUCLEFT-OUT
1	0.74	0.90
2	0.78	0.69
3	0.82	0.61
4	0.81	0.47
5	0.75	0.76

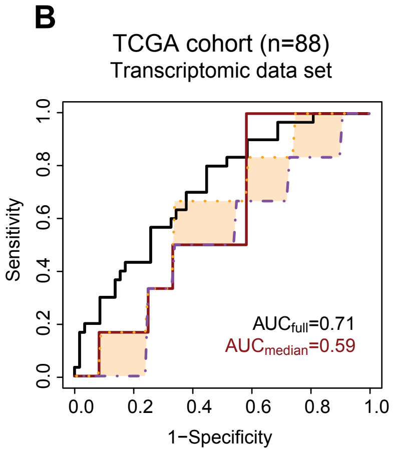

Fold	AUCTRAIN	AUCLEFT-OUT
1	0.73	0.58
2	0.69	0.61
3	0.74	0.59
4	0.73	0.65
5	0.73	0.44

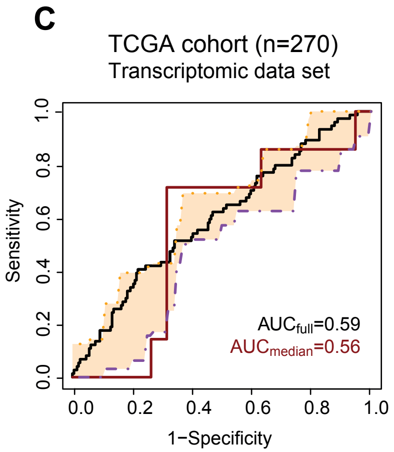

Fold	AUCTRAIN	AUCLEFT-OUT
1	0.61	0.51
2	0.59	0.61
3	0.59	0.55
4	0.59	0.39
5	0.61	0.57
6	0.60	0.50
7	0.58	0.63
8	0.63	0.68
9	0.58	0.56
10	0.61	0.54

Appendix Figure S5

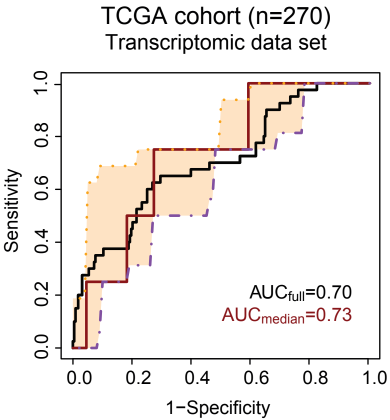

Fold	AUC_{TRAIN}	$AUC_{LEFT-OUT}$
1	0.71	0.63
2	0.68	0.90
3	0.71	0.54
4	0.73	0.61
5	0.73	0.57
6	0.68	0.89
7	0.69	0.75
8	0.73	0.50
9	0.69	0.73
10	0.68	0.88
