# Supplementary material for: Non-invasive prognostic protein biomarker signatures associated with colorectal cancer
Source: EMBO Mol Med. 2015 Aug 7;7(9):1153–65. doi: 10.15252/emmm.201404874 (PMC4568949; doi:10.15252/emmm.201404874)
Supplement: Supplementary file 2 [file emmm0007-1153-sd2.pdf]

Non-invasive prognostic protein biomarker signatures associated with colorectal cancer

Silvia Surinova, Lenka Radová, Meena Choi, Josef Srovnal, Hermann Brenner, Olga Vitek, Marián Hajdúch, Ruedi Aebersold

Corresponding author: Ruedi Aebersold, ETH Zurich

Review timeline:

Submission date:	18 November 2014
Editorial Decision:	09 January 2015
Revision received:	11 May 2015
Editorial Decision:	07 July 2015
Accepted:	07 July 2015

Transaction Report:

(Note: With the exception of the correction of typographical or spelling errors that could be a source of ambiguity, letters and reports are not edited. The original formatting of letters and referee reports may not be reflected in this compilation.)

Editor: Roberto Buccione

1st Editorial Decision 09 January 2015

Thank you for the submission of your two back to back manuscripts EMM-2014-04873, "Prediction of colorectal cancer diagnosis based on circulating plasma proteins" and EMM-2014-04874, "Non-invasive prognostic and predictive protein biomarker signatures associated with colorectal cancer" to EMBO Molecular Medicine. We have now heard back from the three Reviewers whom we asked to evaluate your manuscript.

We are sorry that it has taken longer than usual to get back to you on your manuscript. In this case we experienced some difficulties in securing three appropriate reviewers (due also to the heavier than usual burden), to whom I eventually granted a bit more time than usual. Obviously, the overlap with the holiday season did not help to speed things up.

You will see that the three Reviewers, albeit with varying degrees of enthusiasm, are globally supportive of your work but raise important concerns, which all considered, prevent us from considering publication at this time.

I will not dwell into much detail, and just mention a few main points. In my opinion, two main significant issues emerge, that are consistent across the reviewer panel and valid for both manuscripts. The first is that the clinical relevance of your findings is not convincingly demonstrated and that more experimentation is required to that effect. The second, connected, issue is that the potential advantage of your diagnostic and prognostic approaches with respect to the commonly accepted practices is not shown and must be convincingly explored, not merely discussed. The Reviewers offer many approachable strategies to address these issues experimentally.

To mention one specific point, you will also see that two Reviewers are very critical of the rationale

© EMBO

and usefulness of the KRAS signature in Ms. EMM-2014-04873.

The Reviewers also list other items for both manuscripts, which require your action.

In conclusion, while publication of the paper cannot be considered at this stage, given the potential interest of your findings and the fact that the Reviewers, although critical, were globally positive, we have decided to give you the opportunity to address the above concerns.

We are thus prepared to consider substantially revised submissions, with the understanding that the Reviewers' concerns must be fully addressed with additional experimental data where appropriate and that acceptance of the manuscripts will entail a second round of review. Please note that this should not be considered a commitment to publish on our side and that the two main issues raised as mentioned above, are very important ones for our title and should be satisfactorily addressed for us to consider publication. Also, the final decisions on the two manuscripts will be independent of each other.

Please note that it is EMBO Molecular Medicine policy to allow a single round of revision only and that, therefore, acceptance or rejection of the manuscripts will depend on the completeness of your responses included in the next, final versions of the manuscripts.

As you know, EMBO Molecular Medicine has a "scooping protection" policy, whereby similar findings that are published by others during review or revision are not a criterion for rejection. However, I do ask you to get in touch with us after three months if you have not completed your revision, to update us on the status. Please also contact us as soon as possible if similar work is published elsewhere.

EMBO Molecular Medicine now requires a complete author checklist (http://embomolmed.embopress.org/authorguide#editorial3) to be submitted with all revised manuscripts. Provision of the author checklist is mandatory at revision stage; The checklist is designed to enhance and standardize reporting of key information in research papers and to support reanalysis and repetition of experiments by the community. The list covers key information for figure panels and captions and focuses on statistics, the reporting of reagents, animal models and human subject-derived data, as well as guidance to optimise data accessibility.

I will be sending the same decision letter for both manuscripts so that you will be able to later provide separate rebuttals and revisions.

I look forward to seeing your revised manuscripts in due time.

***** Reviewer's comments *****

Referee #1 (Comments on Novelty/Model System):

In this manuscript the data for the 202 CRC plasma are mined for additional clinical questions. Impact of the prognostic signature may be high when validated in an independent dataset.

Referee #1 (Remarks):

The second paper of the Aebersold group further dissects the targeted MS data generated for the 202 CRC plasma samples already described in paper 1, for the identification of non-invasive prognostic and predictive (KRAs status) signatures for clinical applications in CRC. Again all statistical analyses are solid and clearly described.

My main concern is that the classification performance is reported with internal cross-validation and not yet with an external independent set. Yet the survival curves (Figure 2) shows a very impressive prognostic effect.

The utility of the Kras signature is not so clear.

Why would one want to predict a KRas mutation with a % of certaincy while mutations (this one and other relevant mutations for repsonse to cetuximab like BRaf and NRas mutations) can be sequenced easily in routine practise now? Does this sginature also predict these other clinically relevant mutations?

Referee #2 (Comments on Novelty/Model System):

See comments to authors.

Referee #2 (Remarks):

This manuscript is the further work based on the authors' diagnostic paper. The goal of the study is to develop a biomarker signature predicting the clinical outcome of CRC using a non-invasive approach. The authors employed a target proteomics approach to profile 80 glycoprotein biomarker candidates across a comprehensively characterized CRC cohort. They successfully generated several plasma protein biomarker predictors of overall survival, prognosis, colon versus rectal tumor localization, status of KRAS mutation and metastasis, respectively. They also showed some added value of plasma protein signatures for outcome prediction and stratification of subjects into prognostic groups.

Major Issues:

1. Since the predictors might serve as a prognostic marker of CRC, it will be necessary for the authors to monitor the expression changes of these proteins before and after therapy.

2. The authors should also analyze the mRNA levels of signature proteins using the TCGA or Oncomine database. If part of their proteomics data matches the genomic data, it will definitely strengthen the reliability and impact of their findings.

Referee #3 (Remarks):

In this manuscript the Aebersold laboratory identify potential predictive biomarker signatures for CRC using a mass spec based approach. The discovery data appears robust and sound.
Major points
1. As the authors highlight themselves the key clinical observation in this manuscript is the clear separation of stage 2/3 into good and poor prognosis. However, one of the key shortcomings of this manuscript is that there has been no attempt to put the signatures identified here in the context of recent transcriptional signatures of CRC prognosis and prediction. Last year as exemplified by 2 Nature Medicine papers (one by JP Medema and one by Doug Hanahan) help redefine prognostic signatures in crc. What is the overlap of the proteins identified here. Many of these will be controlled at the RNA level. It should be noted there are already papers looking at individual targets such as CD44 in prognosis.
2. I would describe a predictive signature as one which would predict therapeutic response. Thus im not too sure I agree that this paper is really generating novel predictive signatures. Instead they are trying to see if they can identify signatures that identify the KRAS mutant phenotype and the MSI phenotype. Here they were unable to identify a MSI phenotype, although they had more success in the KRAS phenotype. Again it should be noted there are transcriptionally signature of the "KRAS" mutant CRC. It should also be noted that as sequencing improves then I believe KRAS mutation status will be much more reliably detected on patient material (on both resected material and on circulating DNA) so im not sure if the KRAS mutant signature identified here would be clinically important. Obviously they may be biologically important and known components like KDR have been identified however there is very little biology in this paper so im not sure what this signature

really adds.

Overall given that 2 papers have been submitted, I feel that a single combined study which included data on the early detection and the prognostic signature (e.g.stage 2/3) would make a much stronger paper than the 2 papers separately. However it is vital that the authors at each stage discuss the real likelihood that these markers would be adopted into clinical practice.

1st Revision - authors' response 11 May 2015

Referee #1 (Comments on Novelty/Model System):

In this manuscript the data for the 202 CRC plasma are mined for additional clinical questions. Impact of the prognostic signature may be high when validated in an independent dataset.

Referee #1 (Remarks):

The second paper of the Aebersold group further dissects the targeted MS data generated for the 202 CRC plasma samples already described in paper 1, for the identification of non-invasive prognostic and predictive (KRAs status) signatures for clinical applications in CRC. Again all statistical analyses are solid and clearly described.

My main concern is that the classification performance is reported with internal cross-validation and not yet with an external independent set. Yet the survival curves (Figure 2) shows a very impressive prognostic effect.

We agree with the reviewer that validation of the proposed signatures on an independent dataset is extremely important, and ultimately required to move the markers (if the data are positive) towards clinical use. Unfortunately, it is prohibitively difficult to perform such analyses, at least within the time frame of a paper revision. The current cohort is the result of three years of IRB approval, patient enrolment and sample collection. The recording of the clinical data, and especially of the survival, took five additional years. Moreover, it took us over a year to develop the targeted SRM assays. The multiplexed measurements of the developed assays took over four months, and tailored data analysis followed. It is therefore impossible for us to provide a new independent data set of a similar scale, and using the same measurement technology, during the short revision cycle.

The reviewer's comment highlights a key general problem with protein biomarker studies, i.e. the lengthy time period required to collect and analyse the samples and data. Such large-scale projects quickly become untractable for academic research groups. This is one of the main reasons why the present study is the first of this scale, and as such is unique. We hope that the reviewer agrees with us that, although the present study falls short of independent clinical validation, it represents an advance in the field that is worth reporting. The scope and the quality of these data will serve as a foundation that other researchers can build upon. This is in contrast to many protein biomarker reports where the statistical weight of the reported results is much weaker.

To address the valid concern without the benefit of an independent validation cohort, we sought alternative insights into the predictive ability of the selected signatures. We compiled a collection of three published data sets, all acquired from tumour tissue samples of CRC patients, and related the data of our study to these data. While we did not find any other high quality quantification proteomic data sets with an adequate scope and sample size, the three datasets had measurements with complementary platforms: (i) The TCGA study profiled 270 patients on the transcript level[1]; of these, 90 patients were also profiled by Zhang *et al*. using data-dependent mass spectrometry and semi-quantification by spectral counting[2]; (ii) The GSE14333 dataset from Jorissen *et al*. profiled 289 patients on the transcript level[3]; and (iii) The GSE17536 dataset from Smith *et al*. profiled 177 patients, also on the transcript level[4]. In these cases we used the transcript level as a proxy of the protein abundance. This is certainly an imprecise proxy and we could speculate that the performance would be likely better if protein data were available for the validation cohorts, but this is not the case. We utilized these data sets for the validation of the three proposed signatures in this study as follows:

© EMBO

Prognostic signature of 5 year overall survival. Unfortunately, the TCGA dataset[1] had only limited outcome data, and neither transcriptomic nor proteomic components could be used for predicting the 5 year survival. However, the two other datasets, GSE17536 and GSE14333, contained adequate clinical outcome data. They were also acquired on the same HG-U133Plus2.0 platform. To evaluate our prognostic signature that was developed for patients with localized CRC, we employed patients with stages I-III from the above cohorts. In both cohorts, transcripts coding for all the six proteins in our signature were measured.

In the GSE17536 cohort, 138 out of the 177 patients had TNM stages 1-3 and overall survival was available with a follow up of 12 years. We estimated the parameters of a Cox proportional hazards model, which uses as predictors the genes corresponding to the proteins in our signature. We evaluated the ability of our signature to predict the 5 year OS on the full data set (AUC_{full}=0.76) as well as within 5-fold cross-validation (AUC_{median}=0.61). (NB. As described in the manuscript, here and throughout all discussion AUC_{median} represents an unbiased performance, derived from separate parameter estimation and out-of-sample evaluation of predictive ability within each fold of cross-validation, and calculated as a pseudomedian over the folds. AUC_{full} denotes an upper level of performance, based on the parameter estimation and evaluation of predictive ability on the full data set.)

The GSE14333 cohort did not contain the TNM staging classification, and was associated with 5 years disease-free survival (DFS) (as opposed to the overall survival used in our study). Even though the staging classification and the endpoint are somewhat different, this cohort is relevant to our work. In this cohort 139 of the 289 subjects had Dukes stages A-C, which roughly correspond to non-metastatic stages I-III of the TNM classification. The cohort was followed for a relatively long time (12 years of observations). As above, we estimated the parameters of a Cox proportional hazards model, which uses as predictors the genes corresponding to the proteins in our signature. We evaluated the ability of our signature to predict the 5 year DFS on the full data set (AUC_{full} =0.81) as well as within 10-fold cross-validation (AUC_{median}=0.83).

The classifications obtained on these two cohorts were in accordance with the performance in our cohort (149 of 202 subjects, stage I-III, 5 year OS, AUC_{full} of 0.72, AUC_{median} of 0.75). A somewhat higher performance was obtained when predicting the disease-free survival in the GSE14333 data set as compared to the overall survival in the GSE17536 data set.

Next, we plotted survival curves for individual stages predicted by clinical factors alone, and by clinical factors and the signature genes. This has been done according to the analysis performed on our cohort that is shown in **figure 2b-e**. Similar to the results on the protein level obtained in the present study, both cohorts (GSE17536 and GSE1433) showed a separation of patients into low and high risk groups for all stages when the proxy transcripts of the protein signature were used as predictors. This separation was more pronounced for stage II and III. This pattern was observed for both transcript-based cohorts. Results of these additional analyses have been added to the paper (**figure S2** (GSE17536) and **S3** (GSE14333)).

We hope that these results convinced the reviewer that the proposed biomarker signature is able to stratify subjects with localised CRC for clinical outcome beyond the clinical stage and into high and low risk prognostic groups. Even though the validation was limited to the use of transcriptional data for practical reasons, we expect that the validation at the protein level in an independent cohort would lead to a discrimination that is at least as powerful as the one observed on the transcript level. Furthermore, the independent validation analysis illustrates that high quality clinical cohorts with long survival follow up are unique. These analyses highlight the value of the large prognostic proteomic data set presented in this manuscript for future research.

Localization signature. The clinical annotation of regional localization of CRC as used in the present study was available for the TCGA cohort (colon, n=196, versus rectal, n=74, tumours) and we therefore used these data as an independent validation cohort.

First, we evaluated the predictive ability of our signature on 88 of the 90 patients from the TCGA study, which were also profiled by Zhang *et al.* on the protein level, and which had localization annotation available (colon, n=58, rectum, n=30)[2]. All seven signature proteins in this manuscript were also quantified by Zhang *et al.* Since the nature and precision of semi-quantitative spectral counting data is different from intensity-based SRM quantification data, we had to retrain the parameters of the logistic regression, which used the seven proteins as predictors, on this cohort. We evaluated the ability of the signature to predict colon and rectal localization on the full data set (AUC_{full}=0.78) as well as within 5-fold cross-validation (AUC_{median}=0.69). The results were highly

in accordance with our data, where the classification of 202 patients resulted in the AUC_{full} of 0.75, and AUC_{median} of 0.66.

Since the 90 patients of the TCGA cohort had measurements on both the transcript and the protein level, we could directly examine the level of concordance for specific signatures between the transcriptomic and the proteomic classifications on the same cohort. For the transcriptomic TCGA dataset, we took again the seven signature elements identified in the present study as indirect proxies of protein abundances, trained the parameters of the logistic regression with the seven proteins as predictors. We evaluated the prediction on the full data set (AUC_{full} =0.71) as well as within 5-fold cross-validation (AUC_{median}=0.59). This performance obtained for the transcriptomic TCGA data and the performance obtained for the proteomic TCGA data above (AUC_{full}=0.78; AUC_{median}=0.69) are quite similar but higher on the protein level. Since 90 patients represent a smaller sized cohort, we have further evaluated the transcriptional performance on the full TCGA cohort with 270 patients and found that this trend was more pronounced here (AUC_{full}=0.59, AUC_{median}=0.55).

With these results, we have validated the localization signature on the protein level in an independent, prospectively collected cohort of CRC subjects. Furthermore, on the transcript level we observed a lower performance than on the protein level, suggesting that the signature proteins are regulated to some degree post-translationally. To investigate this for the individual signature proteins, we compared the predictions per protein in our data set and in the complete TCGA cohort on the mRNA level, and confirmed that the classifications were more accurate on the protein than on the transcript level for six out of the seven proteins (FN1 showed a similar performance between the two platforms). The results from these additional analyses have been added to the paper (**figure S4** and **table S14**).

Metastatic signature. The clinical annotation of CRC dissemination as used in the present study was available for the TCGA cohort (localized stages I-III, n=224, versus metastatic stage IV, n=40). However, the 90 patients from the TCGA cohort that were also used by Zhang *et al.*[2] for proteomic measurements had only a limited number of patients in the metastatic group (n=11) as compared to the localised group (n=74). This unbalanced sample size negatively affected the reliability of our evaluation results.

First, we evaluated the metastatic signature on the protein level. This time, only seven of our nine signature proteins were present in Zhang's data. We trained the parameters of the logistic regression model with the reduced signature proteins on these data. We then evaluated the ability of the signature to predict localized and metastatic CRC on the full data set, as well as within 5-fold cross-validation. The performance of the signature in Zhang's data was lower (AUC_{full}=0.74, AUC_{median}=0.56) than in our data (AUC_{full}=0.9, AUC_{median}=0.82). The lower performance could be attributed to some extent to two signature proteins (IGHG2 and FETUB) that were not present in Zhang's data, to the lower accuracy of protein quantification by spectral counting as compared to SRM, and to a low count of metastatic patients in this dataset.

Next, we used the same cohort of 90 patients to examine the metastatic signature on the transcript level. The TCGA transcriptomic data contained genes corresponding to all the nine proteins in our signature. We took these genes as proxies, trained the parameters of the logistic regression with these genes as predictors, and evaluated the classification. The performance was slightly better here (AUC_{full}=0.81, AUC_{median}=0.6) than for the reduced protein signature measured by Zhang. As above, the discrepancy between mRNA and protein-level results for the metastatic signature can be explained in part by a low count of metastatic patients in this dataset.

To examine this last aspect more directly, we evaluated the performance on the mRNA level in the full TCGA data set (270 patients), with 40 subjects in the metastatic group. The logistic regression model was retrained with all the signature genes as above. The classification results showed that the performance in this larger cohort was not as high (AUC_{full}=0.7 and AUC_{median} =0.73) as in the smaller set of 90 patients with the limited number of metastatic cases (AUC_{full}=0.81, AUC_{median}=0.6).

In conclusion, we attempted the validation of the metastatic signature in an independent protein data set. The limitations of this validation were the lower accuracy of quantification by spectral counting, the fact that two of the nine signature proteins were not sampled in this data set, and the fact that the metastatic group was relatively small. By examining the full TCGA data set on the mRNA level we observed that the metastatic signature is regulated to a smaller extent transcriptionally, and to a larger extent post-translationally (as quantified by our data). The results from the analyses on the full TCGA data set have been added to the paper (**figure S5**).

The utility of the Kras signature is not so clear. Why would one want to predict a KRas mutation with a % of certainty while mutations (this one and other relevant mutations for repsonse to cetuximab like BRaf and NRas mutations) can be sequenced easily in routine practise now? Does this sginature also predict these other clinically relevant mutations?

The objective of a signature predicting KRAS mutation was motivated for subjects with samples where the quality of DNA was insufficient for genotyping. A larger study from 2010 reported that 136 of 1022 (13%) tumor samples had insufficient quality DNA and could not be included in mutation frequency analyses[5]. In this study DNA of a sample was considered of sufficient quality when more than 75% of mutations were reliably genotyped. Our proposed solution of the DNA integrity issue was to use a test from the signature proteins in the circulation as a secondary predictor of the KRAS status.

We agree with the reviewer that the technologies for determining the state of these mutations have improved over the recent years, and that there is no longer a significant number of cases with this need. For this reason, we have decided to remove this signature from the manuscript.

Referee #2 (Comments on Novelty/Model System):

See comments to authors.

Referee #2 (Remarks):

This manuscript is the further work based on the authors' diagnostic paper. The goal of the study is to develop a biomarker signature predicting the clinical outcome of CRC using a non-invasive approach. The authors employed a target proteomics approach to profile 80 glycoprotein biomarker candidates across a comprehensively characterized CRC cohort. They successfully generated several plasma protein biomarker predictors of overall survival, prognosis, colon versus rectal tumor localization, status of KRAS mutation and metastasis, respectively. They also showed some added value of plasma protein signatures for outcome prediction and stratification of subjects into prognostic groups.

Major Issues:

1. *Since the predictors might serve as a prognostic marker of CRC, it will be necessary for the authors to monitor the expression changes of these proteins before and after therapy.*

In this manuscript the aim for prognostic markers is to predict outcome and other prognostic features at diagnosis, before any treatment was administered. We agree with the reviewer that monitoring the characterised signatures longitudinally, as well as upon treatment, is valuable. However, it is unfortunately impossible for us to provide these data. Serial samples from the same subjects are currently not available, and would have to be specifically collected *de novo*. The current cohort is the result of three years of patient enrolment and sample collection. The recording of the clinical data, and especially of the survival, took five additional years. The development of the targeted SRM assays took a year and the multiplexed measurements took four months to acquire. It is impossible for us to repeat this within the short review cycle.

As the previous reviewer, the reviewer #2 highlights the key problem in all the protein biomarker studies, i.e. the lengthy time period required to collect and analyse the data. To the best of our knowledge, there is currently no published proteomic dataset of a similar scope and accuracy as the present study, which also contains serial samples. We hope that the reviewer agrees that, although the present study does not have serial measurements, it represents a substantial advance in the field.

To address the reviewer's point at least partially, we examined the abundance changes of the signature proteins between diseased and basal states (i.e. CRC patients versus healthy controls), to comment on the magnitude of change that would need to be reversed by the treatment to return to the normal level. We tested for differential protein abundance between patients of stages I-III and the healthy control cases that we studied in the diagnostic paper. Of the six signature proteins (HLA-A, CFH, CD44, PTPRJ, HP, CDH5), CD44 (FC=1.23, p=4.56e-06), CFH (FC=1.25, p<1e-15), and HP (FC=1.90, p<1e-15) were significantly more abundant in the CRC than in the control group. The other three proteins didn't show a differential abundance between the two groups. We could

speculate that the higher abundance of CD44, CFH, and HP would be expected to return to the level of the normal controls after the treatment. At this point we did not include these analyses in the paper, as we cannot address the reviewer's point of treatment impact adequately without having access to an appropriate sample cohort.

2. The authors should also analyze the mRNA levels of signature proteins using the TCGA or Oncomine database. If part of their proteomics data matches the genomic data, it will definitely strengthen the reliability and impact of their findings.

We thank the reviewer for this suggestion, which we addressed in great detail. A large part of this response also comprises the response to reviewer #1, which we repeat here for completeness. We compiled a collection of three published transcriptomic data sets, all acquired from tumor tissue samples of CRC patients, and related the data of our study to these data. The following three data sets were used: (i) The TCGA study profiled 270 patients on the transcript level[1]; (ii) The GSE14333 dataset from Jorissen *et al*. profiled 289 patients on the transcript level[3]; and (iii) The GSE17536 dataset from Smith *et al*. profiled 177 patients, also on the transcript level[4]. In these cases we used the transcript level as a proxy of the protein abundance. We utilized these data sets for the validation of the proposed outcome, regional localization and metastatic dissemination signatures as follows:

Prognostic signature of 5 year overall survival. Unfortunately, the TCGA dataset had only limited clinical outcome data and could not be used for predicting the 5 year survival. However, the two other datasets, GSE17536 and GSE14333, contained adequate clinical outcome data. They were also acquired on the same HG-U133Plus2.0 platform. To evaluate our prognostic signature that was developed for patients with localized CRC, we employed untreated patients with stages I-III from the above cohorts. In both cohorts, transcripts coding for all the six proteins in our signature were measured.

In the GSE17536 cohort, 138 out of the 177 patients had TNM stages 1-3 and overall survival was available with a follow up of 12 years. We estimated the parameters of a Cox proportional hazards model, which uses as predictors the genes corresponding to the proteins in our signature. We evaluated the ability of our signature to predict the 5 year OS on the full data set (AUC_{full}=0.76) as well as within 5-fold cross-validation (AUC_{median}=0.61). (NB. As described in the manuscript, here and throughout all discussion AUC_{median} represents an unbiased performance, derived from separate parameter estimation and out-of-sample evaluation of predictive ability within each fold of cross-validation, and calculated as a pseudomedian over the folds. AUC_{full} denotes an upper level of performance, based on the parameter estimation and evaluation of predictive ability on the full data set.)

The GSE14333 cohort did not contain the TNM staging classification, and was associated with 5 years disease-free survival (DFS) (as opposed to the overall survival used in our study). Even though the staging classification and the endpoint are somewhat different, this cohort is relevant to our work. In this cohort 139 of the 289 subjects had Dukes stages A-C, which roughly correspond to non-metastatic stages I-III of the TNM classification. The cohort was followed for a relatively long time (12 years of observations). As above, we estimated the parameters of a Cox proportional hazards model, which uses as predictors the genes corresponding to the proteins in our signature. We evaluated the ability of our signature to predict the 5 year DFS on the full data set (AUC_{full}=0.81) as well as within 10-fold cross-validation (AUC_{median}=0.83).

The classifications obtained on these two cohorts were in accordance with the performance in our cohort (149 of 202 subjects, stage I-III, 5 year OS, AUC_{full} of 0.72, AUC_{median} of 0.75). A somewhat higher performance was obtained when predicting the disease-free survival in the GSE14333 data set as compared to the overall survival in the GSE17536 data set.

Next, we plotted survival curves predicted by stage alone, and by stage and the signature genes. This has been done according to the analysis in our cohort (**figure 2b-e**). Similar to the results on the protein level obtained in the present study, both cohorts (GSE17536 and GSE1433) showed a separation of patients into low and high risk groups for all stages when the proxy transcripts of the protein signature were used as predictors. This separation was getting more pronounced for stage II and III. This pattern was observed for both transcript-based cohorts. Results from these additional analyses have been added to the paper (**figure S2** (GSE17536) and **S3** (GSE14333)).

We hope that these results convinced the reviewer that the proposed biomarker signature is able to stratify subjects with localised CRC for clinical outcome also at the transcript level. Furthermore,

© EMBO

the independent validation analysis illustrates that high quality clinical cohorts with long survival follow up are unique. These analyses highlight the value of the large prognostic proteomic data set presented in this manuscript for future research.

Localization signature. The clinical annotation of regional localization of CRC as used in the present study was available for the TCGA cohort (colon, n=196, versus rectal, n=74, tumours) and we therefore used these data as an independent validation cohort. From this data set, we took the seven signature elements identified in the present study as indirect proxies of protein abundances, estimated the parameters of a logistic regression model, and evaluated the prediction on the full data set (AUC_{full}=0.59) as well as within 10-fold cross-validation (AUC_{median}=0.55).

On the transcript level we observe a lower performance than on the protein level in our data set (AUC_{full} =0.75, AUC_{median}=0.66.) suggesting that the signature proteins are regulated to some degree post-translationally. To investigate this for the individual signature proteins, we compared the predictions per protein in our data set and in the TCGA cohort on the mRNA level, and confirmed that the classifications were more accurate on the protein than on the transcript level for six out of the seven proteins (FN1 showed a similar performance between the two platforms). The results from these analyses have been added to the paper (**figure S4c** and **table S14**).

Metastatic signature. The clinical annotation of CRC dissemination as used in the present study was available for the TCGA cohort (localized stages I-III, n=224, versus metastatic stage IV, n=40).

To examine the metastatic signature on the transcript level, we took the nine signature proteins as proxies, trained the parameters of the logistic regression model with these genes as predictors, and evaluated the prediction ability. The obtained classification showed that the performance on the mRNA level (AUC_{full}=0.7 and AUC_{median}=0.73) was not as high as on the protein level (AUC_{full}=0.9, AUC_{median}=0.82). In conclusion, by examining the transcriptomic TCGA data set we observed that the metastatic signature is regulated to a lesser degree transcriptionally and to a much larger degree post-translationally (as reported by our data). The results from these analyses have been added to the paper (**figure S5**).

Referee #3 (Remarks):

In this manuscript the Aebersold laboratory identify potential predictive biomarker signatures for CRC using a mass spec based approach. The discovery data appears robust and sound.

Major points

1. As the authors highlight themselves the key clinical observation in this manuscript is the clear separation of stage 2/3 into good and poor prognosis. However, one of the key shortcomings of this manuscript is that there has been no attempt to put the signatures identified here in the context of recent transcriptional signatures of CRC prognosis and prediction. Last year as exemplified by 2 Nature Medicine papers (one by JP Medema and one by Doug Hanahan) help redefine prognostic signatures in crc. What is the overlap of the proteins identified here. Many of these will be controlled at the RNA level. It should be noted there are already papers looking at individual targets such as CD44 in prognosis.

Unlike the previous reviewers, who asked us to evaluate the performance of our prognostic signature on independent datasets, this reviewer asks us to compare our protein signature to transcriptional signatures proposed by others that were derived from tumor tissue measurements. We agree with the reviewer that this comparison is important. We examined the classifiers reported by De Sousa E Melo *et al*.[6] (the 'Medema manuscript', employing the GSE33113 data set[7]), and by Sadanandam *et al*.[8] (the 'Hanahan manuscript', employing GSE13294[9] and GSE14333[3] data sets), and the ability of our protein signature to predict their prognostic endpoints.

De Sousa E Melo *et al*. characterised three molecularly distinct colon cancer subtypes (CCSs) in a cohort of 90 stage II patients. Patients predicted to represent the CCS3 subtype demonstrated the worst prognosis. Sadanandam *et al*. defined five prognostic subtypes related to cellular phenotypes, and developed a classifier to predict these subtypes. Two of these subtypes (transit-amplifying and goblet-like subtype) showed good prognosis, two subtypes (inflammatory and enterocyte subtype) showed intermediate prognosis, and the stem-like subtype demonstrated the worst prognosis. Two of

the data sets (GSE33113 and GSE14333) were associated with survival data, specifically with disease-free survival (DFS), as opposed to overall survival (OS) in our manuscript. Since the data sets used in the two Nat Med papers were associated with DFS instead of OS, to address the first part of the comment we also considered an additional appropriate data set (GSE17536 from Smith *et al.*[4]), which used OS as the clinical endpoint.

We first addressed the comments regarding (i) the prognostic value of individual proteins from our signature on the protein and transcript level, (ii) any prior knowledge for these proteins, and (iii) any overlap between our outcome signature and the transcriptional signatures of the two Nat Med papers.

(i) To evaluate the individual performances in the two transcriptomic datasets introduced above, we trained the parameters of six Cox proportional hazards models, each using the expression values of one of the six genes as a predictor. We then reported the performance for the full data, as well as after training the parameters and evaluating the predictive performance within cross validation in each of the five folds in the case of GSE17536 and of the ten folds in the case of GSE14333 data. The transcript abundance was used as proxy of the signature protein abundance. When comparing the areas under the ROC curves of individual proteins (from our data) and genes (from these two mRNA data), only CD44 and PTPRJ on the protein level (in our data) and CFH on the transcript level (in GSE17536 data) showed higher areas than 0.6. We followed the same procedure for the GSE14333 data associated with DFS (used in Sadanandam *et al.*) and found again that only CFH showed a higher area than 0.6 on the transcript level. The results of these comparisons are represented in **table S11**.

(ii) In our literature search of prior prognostic evidence of the proposed signature proteins, we only found a report for CD44 where tumour specimens from 74 patients were assayed by immunohistochemistry[10]. We believe that an accurate mass spectrometry-based quantification of CD44 in plasma, across a large cohort and for the purposes of prognostics as performed in our manuscript, is novel. Moreover, our data indicate that the multivariate nature of the proposed signature contributes greatly to its performance as compared to the performance of the individual signatures. The latter can be seen by comparing the predictive performance of the individual signature proteins (in our proteomic data), or individual gene proxies (in transcriptomic data), summarized in (i), to the performance of the multivariate signature. We have added the CD44 discussion into the manuscript.

(iii) Finally, we examined the overlap between the genes of the transcriptional signatures in these two manuscripts and the proteins in our signature. While the manuscripts profiled intracellular and signalling proteins, as done in most studies of CRC biology in tissues, our study focused on blood circulation. We therefore do not expect a large overlap. Indeed, CFH was the only overlapping molecular entity, it was present in the 146-gene signature of De Sousa E Melo *et al.* as well as in our study. The 30-gene signature of Sadanandam *et al.* had no overlap with our signature. However, the initial classifier developed by Sadanandam *et al.*, which was comprised of 786 subtype-specific signature genes and was later condensed into the 30-gene signature, also contained CFH.

Next, we examined the ability of our signature to predict the clinical endpoints in the two manuscripts. For the De Sousa E Melo *et al.* manuscript that characterized three molecularly distinct colon cancer subtypes (CCSs), we investigated our ability to predict any of these subtypes. We used the 90 patients in the GSE33113 dataset, and a multinomial regression model with the six gene proxies from our signature as predictors. We used 10-fold cross-validation to train the parameters of the multinomial regression in each fold, and to perform the out-of-sample evaluation of the predictive ability. We were able to accurately predict 75% of cases with CCS1, 33% of cases with CCS2, and 83% of cases with CCS3. The Kaplan-Meier survival curves of the three subtypes obtained from our classification were highly in agreement with the curves in De Sousa E Melo *et al.* These results demonstrated that the proposed signature can predict patients with good and bad prognosis (i.e., subtype CCS1 and CCS3) particularly well, and that this prediction can be achieved using a minimally invasive procedure from the circulation by measuring six proteins. These analyses are now part of **figure 4a** and **table S12,** which details the prediction tables from cross validation.

For Sadanandam *et al.*, we examined the ability of the proposed signature to predict the five prognostic subtypes related to cellular phenotypes. To this end we trained the parameters of a

© EMBO

multinomial model with the six gene proxies from our signature as predictors and classified the patient samples from two data sets (GSE13294 data contained 135 patients and GSE14333 data contained 152 patients) in each fold of cross validation (5 and 10 folds were used for GSE13294 and GSE14333, respectively). In both datasets we could most accurately classify the transit-amplifying subtype, demonstrating good prognosis. Moreover, in the GSE14333 data set, the stem-like (i.e. bad prognosis) subtype could be predicted with a median correct classification percentage of 67%. Since GSE14333 contained DFS follow up, we plotted the Kaplan-Meier survival curves based on our classification and could reproduce the outcome ranking (i.e. best to worst survival time) for all five subtypes. Details of all these analyses are part of **figure 4b** and **table S12.**

We would like to note some critical differences between the signatures of the two papers above, and our proposed protein signature. First, the existing manuscripts take as input measurements of transcriptional regulation. In contrast, in our study we use post-translational regulation of glycoproteins. Second, the manuscripts take as input the amount of mRNA measured in the tumor tissue, while the present study quantifies the amount of glycoproteins secreted into the circulation. Our choice was made with a clinical utility in mind, and aimed to facilitate the development of non-invasive prognostic techniques. Third, while the classifiers in these manuscripts are quite complex (146 genes in De Sousa E Melo *et al*., and 30 genes in Sadanandam *et al*.), our proposed signature is more concise. It is comprised of six proteins.

With the above analyses we showed that our outcome signature holds prognostic value also on the mRNA level, and was able to predict certain prognostic subtypes of CRC recently defined by the two gene expression signatures.

2. I would describe a predictive signature as one which would predict therapeutic response. Thus im not too sure I agree that this paper is really generating novel predictive signatures. Instead they are trying to see if they can identify ***signatures that identify the KRAS mutant phenotype and the MSI phenotype****. Here they were unable to identify a MSI phenotype, although they had more success in the KRAS phenotype.*

Again it should be noted ***there are transcriptionally signature of the "KRAS"mutant CRC****. It should also be noted that as sequencing improves then I believe KRAS mutation status will be much more reliably detected on patient material (on both resected material and on circulating DNA) so im not sure if the KRAS mutant signature identified here would be clinically important. Obviously they may be biologically important and known components like KDR have been identified however there is very little biology in this paper so im not sure what this signature really adds.*

The motivation behind the signature predicting KRAS mutation on the protein level in the circulation was motivated for samples with insufficient DNA quality for genotyping. A larger study from 2010 reported that 136 of 1022 (13%) tumor samples had insufficient DNA quality and had to be omitted from mutation frequency analyses[5]. Our proposed solution to this issue was to use a test from the signature proteins in the circulation as a secondary predictor of the KRAS status. In this way, some of the samples could be rescued and the test could provide the necessary tailored treatment solutions for the affected patients.

We agree with the reviewer that the technologies for determining the state of these mutations have improved over the recent years and that there is no longer a significant number of cases with this need. Since this point was raised by two reviewers, for the above reason we decided to remove the KRAS signature from the manuscript.

Overall given that 2 papers have been submitted, I feel that a single combined study which included data on the early detection and the prognostic signature (e.g.stage 2/3) would make a much stronger paper than the 2 papers separately. However it is vital that the authors at each stage discuss the real likelihood that these markers would be adopted into clinical practice.

The present study was designed to develop biomarker signatures for prognostic stratification of CRC, which would be comprised of a handful of proteins to facilitate their measurement in a clinical setting. The proposed approach quantifies the amount of glycoproteins secreted into the circulation. We made this choice to translate protein biomarker candidates from tissue to the blood circulation, and to facilitate non-invasive prognosis techniques. Therefore, the proposed signature has a greater potential to be translated into the clinic where a small set of markers can be assayed from a blood sample.

We do realize of course, and discuss extensively, the need for validation in independent cohorts. We do not suggest that the signatures are ready for clinical use at this point. However, we do

suggest that the signatures have a demonstrated level of validation and robustness to warrant publication, also to serve as the base for further studies. Compared to other biomarker reports, this study is a big step forward. The careful selection of the biomarker candidates, which are more realistic for clinical use, is very important. The large sample size is important and very unique in the area of protein data sets. The development of SRM assays is also a big plus, because there is a clear path towards understanding the technological properties of these assays – precision of protein analyte identification and accuracy of analyte quantification, as we used internal standards for each analyte. Protein analytes with interferences were removed during the assay development stage. Also, the fact that the results were so well reproduced across multiple transcriptomic studies is encouraging, and gives us more confidence that the results are not an artifact. All in all, we can say that, while we cannot exactly predict what will happen in the future, this study has a number of very strong characteristics that will encourage or facilitate the transition into the clinic.

Ultimately, only further clinical evaluation will determine the clinical utility of the prognostic signatures reported here and factors (e.g. analysis cost, market size,) beyond the analytical performance will contribute to the implementation of the signature-based test in the clinic. A lot of further work – on the scientific and commercial front – needs to be accomplished to bring the signatures closer to the clinic.

© EMBO

References

1 Cancer Genome Atlas, N. Comprehensive molecular characterization of human colon and rectal cancer. *Nature* **487**, 330-337, doi:10.1038/nature11252 (2012).

2 Zhang, B. *et al.* Proteogenomic characterization of human colon and rectal cancer. *Nature* **513**, 382-387, doi:10.1038/nature13438 (2014).

3 Jorissen, R. N. *et al.* Metastasis-Associated Gene Expression Changes Predict Poor Outcomes in Patients with Dukes Stage B and C Colorectal Cancer. *Clin Cancer Res* **15**, 7642-7651, doi:10.1158/1078-0432.CCR-09-1431 (2009).

4 Smith, J. J. *et al.* Experimentally derived metastasis gene expression profile predicts recurrence and death in patients with colon cancer. *Gastroenterology* **138**, 958-968, doi:10.1053/j.gastro.2009.11.005 (2010).

5 De Roock, W. *et al.* Effects of KRAS, BRAF, NRAS, and PIK3CA mutations on the efficacy of cetuximab plus chemotherapy in chemotherapy-refractory metastatic colorectal cancer: a retrospective consortium analysis. *Lancet Oncology* **11**, 753-762, doi:Doi 10.1016/S1470-2045(10)70130-3 (2010).

6 De Sousa, E. M. F. *et al.* Poor-prognosis colon cancer is defined by a molecularly distinct subtype and develops from serrated precursor lesions. *Nature medicine* **19**, 614-618, doi:10.1038/nm.3174 (2013).

7 de Sousa, E. M. F. *et al.* Methylation of cancer-stem-cell-associated Wnt target genes predicts poor prognosis in colorectal cancer patients. *Cell stem cell* **9**, 476-485, doi:10.1016/j.stem.2011.10.008 (2011).

8 Sadanandam, A. *et al.* A colorectal cancer classification system that associates cellular phenotype and responses to therapy. *Nature medicine* **19**, 619-625, doi:Doi 10.1038/Nm.3175 (2013).

9 Jorissen, R. N. *et al.* DNA Copy-Number Alterations Underlie Gene Expression Differences between Microsatellite Stable and Unstable Colorectal Cancers. *Clin Cancer Res* **14**, 8061-8069, doi:Doi 10.1158/1078-0432.Ccr-08-1431 (2008).

10 Huh, J. W. *et al.* Expression of standard CD44 in human colorectal carcinoma: association with prognosis. *Pathology international* **59**, 241-246, doi:10.1111/j.1440-1827.2009.02357.x (2009).

2nd Editorial Decision 07 July 2015

Please find enclosed the final reports on your manuscript. We are pleased to inform you that your manuscript is accepted for publication and is now being sent to our publisher to be included in the next available issue of EMBO Molecular Medicine.

Congratulations on your interesting work,

***** Reviewer's comments *****

Referee #1 (Remarks):

My comments have been addressed. In particular, the added data mining has strengthened the paper and provides relevant context to the findings.

Referee #2 (Remarks):

The authors have addressed the concerns raised in the first review. They have used two GEO datasets to validate the prognostic value of their biomarker signature, which made a significant improvement over the earlier version of the manuscript.

Referee #3 (Remarks):

The authors have done much in the revision process to compare their protein signatures with the currently available transcriptional signatures.
In their rebuttal letter they have addressed my remaining concerns.
At first I was unsure that the 2 manuscripts submitted were strong enough for 2 independent manuscripts however the extra work done in revision but given this extra work I feel now that they will be an important contribution to the literature.
